# Mental health and empowerment of university students: mediating role of mental health awareness

**DOI:** 10.1186/s40359-025-03726-5

**Published:** 2025-12-04

**Authors:** Mariam Abbas Soharwardi, Razia Anjum, Anna Rana, Javeria Sarwar, Sarmad Rahat, Summayya Waseem, Muhammad Saeed Iqbal

**Affiliations:** 1 Department of Economics, The Islamia University of Bahawalpur, Bahawalpur, Pakistan; 2 Department of Psychology, Bath Spa University, Academic Centre, Ras al Khaimah, United Arab Emirates; 3 University College of Nursing, The Islamia University of Bahawalpur, Bahawalpur, Pakistan; 4 Department of Economics, Lahore College for Women University, Lahore, Pakistan; 5 Department of Economics, Shah Abdul Latif University Khairpur, Khairpur, Pakistan; 6 Lahore School of Behavioural Sciences, University of Lahore, Sargodha Campus, Sargodha, Pakistan; 7 Islamic Business School, Universiti Utara Malaysia, Kuala Lumpur, Malaysia

**Keywords:** Mental health, Empowerment, Mental health awareness, University students, PLS-SEM, Pakistan

## Abstract

The Present study explores students’ mental health and empowerment, focusing on the mediating role of mental health awareness. Methods: Using Partial Least Squares Structural Equation Modeling (PLS-SEM), the research investigates how parental education, parental behavior, self-esteem, self-management, and social media impact mental health through mental health awareness. Data from 350 university students were collected through an online survey. Results: Our findings reveal that mental health awareness significantly influences mental health (β = 0.595, *p* < 0.001). Empowerment, particularly self-esteem (β = 0.212, *p* = 0.003) and self-management (β = 0.620, *p* < 0.001), plays a vital role in improving mental health through increased awareness. Parental behavior also emerged as a significant factor (β = 0.193, *p* < 0.001). However, the influence of parents’ education and financial issues showed less significant effects. Conclusion: The study concludes that mental health awareness is central to improving mental health outcomes, while empowerment and parental behaviors are vital mediating factors. These findings contribute to the understanding of how youth mental health can be enhanced through both internal (empowerment) and external (parental influence) factors.

**Supplementary Information:**

The online version contains supplementary material available at 10.1186/s40359-025-03726-5.

## Introduction

Mental illness is one of the most prevailing medical disorders that become the reason to depression and disabilities among young students Depression is becoming very common among university students due to various reasons in both developing as well as developed countries. Students who take admission in a university come from different socio-economic backgrounds, which may lead to various mental health issues. In Pakistani universities, some students are domestic residents, while others are from different cities and towns [1]. The main purpose of this paper is to explore the risk factors that are involved in students’ mental health issues and to present some solutions to tackle this severe phenomenon. Prevailing research demonstrates six different themes of risk factors that are social, financial, lifestyle, biological, academic, and psychological. Each risk factor has a different intensity in affecting the stress and depression level of university students.

In the long term, if the issue remains unsolved, then it may lead to persistent adverse consequences for young adults; consequently, by the age of 25, they may suffer their first mental illness [2]. Throughout some previous decades, researchers documented numerous areas in mental health research that could be suitably tackled, and consequently, there may be considerable deterioration in the prevalence of mental disorders among the young population. Prevention, care, and promotion are areas that are classified as the focus for future research by adopting some strategies that should be evaluated and followed up.

One more strategy could be the formation of effective resources of evidence that are certainly available through old-fashioned broadcasting, social mass media, and virtual communication components [3]. Requirements aimed at various kinds of economic and well-organized mental health anticipation plans were organized in the collected work [4] Avoidance has been observed utilizing a pre-emotive method to mental health for the reason that it can alleviate threat elements as well as boost spirit in adolescent persons. An evolving topic inside previous writings remains that mental healthiness anticipation may raise equally an optimistic and pessimistic response amongst scholars [5] Once observed optimistically, mind disease deterrence is taken using capital spending in the forthcoming of Canadian families and childhood. On the other hand, once observed in a pessimistic way, avoidance is realized as an original choice. Students whose mental illnesses are explained by an abstract syndrome are justified in believing that their illness was inevitable and could not have been prevented by any action or intervention.

American studies [6–8] lead to the conclusion that the last few decades saw a decline in the psychological health of adults and traumatized students in universities. The authors studied the groups that reported levels of emotional and stress-related problems were higher than their younger counterparts. Stallman studied [9] university counselling services and found increasing numbers of students over the past five years who presented with serious psychological problems. Stallman and Shochet [10] also showed that university students have a higher degree of stress than age-matched nonstudents. Hunt and Eisenberg [11] confirmed this through a national study in which 95% of directors of college counselling services reported an upsurge in students with severe mental issues.

Mewett and Sewyer [12] investigated the mental health of youth worldwide as they explored that tertiary level students are facing more stress issues than the general population, and international students are facing more anxiety issues than domestic undergraduates at a cluster of eight (Go8) Australian universities. Such as with what their families, peers, societal systems, and religious connections have referenced, undergraduate students’ approaches and perspectives on mental well-being are likely to draw from multiple sources [13–17]. The community holds an approach to mental health formed by these personal approaches of undergraduates for mental health [18]. Literature has shown that there are some inappropriate observations between the principles and approaches of the normal society and people supported by mental health professionals, and that many symbols and signs of mental illness are commonly neglected by society [19]. Traditionally, the major community in the main has perceived psychotherapeutic methods to be destructive - prescription, hospitalization, etc. The studies recommend improvement at a general level in mental health numeracy, yet there is still potential for advancement [20]. As mental health literacy could contribute to shaping perceptions and attitudes about mental illnesses, which then affects the development of amateur assessments or amateur analysis [21]. There is evidence that more than 2/3 of university students who could recognize some sort of ailment were not under proper treatment [22]. Trials have mostly been described by young university students in acquiring current operational and sizeable agendas for mental health assistance as constraints for their help-seeking behaviors [23].

As mental health literacy could contribute to shaping perceptions and attitudes about mental illnesses, which then affects the development of amateur assessments or amateur analysis [21]. There is evidence that more than two-thirds of university students who could recognize some sort of ailment were not under proper treatment [22]. Trials have mostly been described by young university students in acquiring current operational and sizeable agendas for mental health assistance as constraints for their help-seeking behaviors [23].

The current literature review provides a general overview of student mental health but is outdated and relies heavily on studies before 2020, limiting its relevance to contemporary contexts. Recent empirical research from 2020 to 2025 highlights the evolving challenges and interventions in student mental health, particularly in Asian countries. For instance, Lee et al. [24] emphasized the critical role of mental health literacy in enhancing students’ awareness and coping strategies, showing that knowledge significantly mediates stress reduction. Similarly, Shibuya [25] compared school-based mental health education in the Philippines, Indonesia, and Japan, revealing that culturally tailored curricula—such as integrating mental health topics in health, values, or religious education—effectively enhance awareness and reduce stigma. Furthermore, studies in India report alarming trends: over 60% of students experience sleep difficulties, 70% face concentration problems, and 6–10% exhibit high psychological distress requiring urgent intervention [26]. Peer-support initiatives, such as India’s “Beacon Buddies” program, have demonstrated effectiveness in empowering students through emotional support and early identification of stress and anxiety.

Mental health challenges among university students are a growing concern globally, with rising prevalence of depression, anxiety, and stress reported in both developing and developed countries [27, 28]. In Pakistan, students’ diverse socio-economic backgrounds, relocation for higher education, and adjustment to new social environments contribute to increased vulnerability to psychological disorders [29]. Previous studies identify multiple risk factors, including social pressures, academic workload, financial constraints, lifestyle habits, and family dynamics, each influencing mental well-being differently [30, 31]. Awareness of mental health issues is crucial for early recognition and help-seeking behavior among youth [32]. Empowerment constructs such as self-esteem and self-management have been linked to improved coping strategies and reduced depressive symptoms [33, 34]. Parental behavior, including emotional support and monitoring, significantly affects adolescent mental health outcomes [35]. Additionally, social media, while providing informational and social support, may both positively and negatively impact mental health and decision-making empowerment, warranting careful examination [36].

Mental health challenges among university students are a growing concern globally, with rising prevalence of depression, anxiety, and stress reported in both developing and developed countries. In Pakistan, students’ diverse socio-economic backgrounds, relocation for higher education, and adjustment to new social environments contribute to increased vulnerability to psychological disorders [28, 37]. Previous studies identify multiple risk factors, including social pressures, academic workload, financial constraints, lifestyle habits, and family dynamics, each influencing mental well-being differently [29]. Awareness of mental health issues is crucial for early recognition and help-seeking behavior among youth. Empowerment constructs such as self-esteem and self-management have been linked to improved coping strategies and reduced depressive symptoms. Parental behavior, including emotional support and monitoring, significantly affects adolescent mental health outcomes [28, 30–38]. Additionally, social media, while providing informational and social support, may both positively and negatively impact mental health and decision-making empowerment, warranting careful examination.

The current study portrays the awareness level of university students concerning their mental health and explores factors leading to their mental illness, along with the possible mediating effects of awareness about mental health and empowerment. It is a novel contribution to understanding the dynamics between mental health, youth empowerment, and mental health awareness. Unlike prior studies that typically focus on one or two factors influencing mental health outcomes, this research integrates a comprehensive model combining empowerment, parental behavior, and digital platforms. By using Partial Least Squares Structural Equation Modeling (PLS-SEM), it expands on existing frameworks by incorporating the mediating role of mental health awareness and exploring indirect effects through empowerment variables such as self-esteem and self-management. This approach provides a more holistic perspective compared to earlier studies, which mainly focused on isolated factors like parental education or financial stress. Therefore, the study not only extends but also challenges existing theories by considering a broader set of influencing factors and their interrelationships in a university context, making a significant contribution to both theoretical and practical advancements in mental health promotion among youth.

While the study claims novelty, the distinction from prior research is not clearly articulated. Most existing studies on student mental health focus on isolated factors such as financial stress, parental influence, or individual coping strategies [44–45[, without integrating these elements into a comprehensive framework. This study’s novelty lies in its multi-dimensional model that simultaneously examines empowerment, parental behavior, digital engagement, and mental health awareness, providing a holistic perspective on the determinants of university students’ mental well-being. Unlike earlier frameworks, it explicitly incorporates the mediating role of mental health awareness, highlighting indirect pathways through empowerment variables such as self-esteem, self-management, and decision-making capacity. Furthermore, the study applies Partial Least Squares Structural Equation Modeling (PLS-SEM) to explore complex interrelationships, which allows for testing both direct and indirect effects—a methodological advancement over traditional regression-based studies. By integrating these multiple layers of influence, the research not only extends theoretical understanding but also offers practical insights for interventions in higher education settings, making it distinct and valuable compared to prior works.

## Methodology

### Conceptual model

PLS-SEM was applied to investigate mental health awareness, the various factors affecting mental health, and the conceptual framework per the behavior theories espoused by noted psychiatrists [11]. The behavior theories emphasized not only people’s inner emotional and psychological states but also considered the external and outward behaviors. External environment and physical responses affect internal feelings because human behavior always follows external and environmental factors. This study emphasizes the need to study factors external to the individual and factors relating to human behavior that impact the mental health profile of youth from the perspective of university students. Mental health has continued to be one of the neglected areas in developing countries.

The SDGs present a clear agenda for health and well-being, which cannot be achieved unless mental health, learning, education, and early child development issues are fast-tracked for youth. Previous studies highlight that there exists a gap between mental health awareness and mental health. In this study, different dimensions of youth empowerment will be used to establish a very strong linkage between empowerment and mental health, with the mediation effects of mental health awareness. Using SEM, a model was designed, and a total of 34 most profound factors were identified from the literature to be observed variables. Nine groups of latent variables were constructed: Mental Health, Mental Health Awareness, Financial Issues, Parent Education, Parent Behavior, social media, Empowerment-Decision Making, Empowerment-Self-Esteem, and Empowerment-Self-Management.

#### Direct hypotheses

- H1: Mental health awareness has a positive and significant effect on mental health.
- H2: Parents’ education has a positive and significant effect on mental health awareness.
- H4: Parents’ behavior has a positive and significant effect on mental health awareness.
- H6: Empowerment (self-esteem) has a positive and significant effect on mental health awareness.
- H7: Empowerment (self-esteem) has a positive and significant effect on mental health.
- H10: Social media has a positive and significant effect on mental health.
- H11: Social media has a negative and significant effect on empowerment (decision making).
- H13: Empowerment (decision making) has a positive and significant effect on mental health
- H14: Financial issues have a negative and significant effect on mental health.
- H15: Empowerment (self-esteem) has a positive and significant effect on mental health.

#### Indirect (Mediated) hypotheses

- H3: Parents’ education has a positive and significant effect on mental health, with the mediating role of mental health awareness.
- H5: Parents’ behavior has a positive and significant effect on mental health, with the mediating role of mental health awareness.
- H8: Empowerment (self-esteem) has a positive and significant effect on mental health, with the mediating role of mental health awareness.
- H9: Empowerment (self-management) has a positive and significant effect on mental health, with the mediating role of mental health awareness.
- H12: Social media has a negative and significant effect on mental health, with the mediating role of empowerment (decision making).

To strengthen novelty refine social-media hypotheses — replace H11–H13 with theoretically grounded, mechanism-specific propositions (e.g., social media → informational/social comparison pathways → decision-making self-efficacy → mental health), supported by recent empirical work; For H11–H13, explicitly ground social-media effects in contemporary empirical work (e.g., information exposure vs. social comparison mechanisms) and specify whether social media fosters or undermines decision-making via moderation/mediation; split social media into positive (informational/support) and negative (comparison/misinformation) facets. Combine overlapping self-esteem hypotheses (H6–H8) into a parsimonious pathway—e.g., self-esteem → mental health awareness → mental health—to avoid redundancy.

#### Theoretical justification

PLS-SEM was used to analyze the relationships between youth empowerment, mental health, and mental health awareness. The hypotheses are grounded in established theories, including the Theory of Planned Behavior (TPB) for empowerment, Social Cognitive Theory (SCT) for self-esteem, and the Health Belief Model (HBM) for mental health awareness. Previous studies have shown that mental health awareness can significantly influence mental health outcomes, while parental factors such as education and behavior are critical in shaping youth mental health awareness [39]. Empowerment, especially in decision-making and self-management, is known to positively affect youth well-being [40]. Financial stress is consistently linked to poorer mental health outcomes [41], while social media’s complex influence on youth empowerment and mental health has been widely documented [42]. PLS-SEM will allow us to validate these relationships and explore the mediating effects of mental health awareness.

The link between **“**Mental Health Awareness” and “Mental Health” is formed by the Health Belief Model (HBM), which suggests that an individual’s knowledge and awareness of health issues influence their attitudes and actions toward preventive and treatment behaviors [43]. Increased awareness can reduce stigma and improve help-seeking behaviour, ultimately enhancing mental health. Parental Education and Behavior, formulated by Ecological Systems Theory [44], the family is a central micro-system influencing a child’s development. Parents’ education and behavior play a significant role in shaping the health beliefs and awareness of their children, including mental health literacy. Studies confirm this pathway by demonstrating the role of parental support and modeling in youth mental health outcomes. The Financial Stress and Mental Health hypothesis is grounded in the Family Stress Model [45], which identifies economic hardship as a major stressor that contributes to psychological distress and negative emotional outcomes in individuals. Chronic financial strain reduces psychological resources, leading to anxiety and depression. Social Media Use linked with Youth Empowerment and Mental Health is based on the Uses and Gratifications Theory and SCT, Social media can both empower (through identity exploration, social connection, and information access) and harm (through cyberbullying, comparison, and screen fatigue) youth mental health. By grounding each relationship in these theoretical frameworks and empirical studies, the model gains robust conceptual support. PLS-SEM is thus appropriately used to validate the measurement model and test the structural pathways, including mediating effects such as that of mental health awareness between empowerment and mental health.

Social Cognitive Theory (SCT) and the Theory of Planned Behavior (TPB) — both offering robust explanatory power for understanding students’ empowerment, awareness, and behavioral intentions toward mental health. Each hypothesis is now directly and explicitly linked to these frameworks, ensuring theoretical coherence and relevance.

The SCT is applied to explain how self-efficacy, parental influence, and social modeling (via social media and peer environments) shape students’ empowerment and awareness behaviors. Meanwhile, TPB supports the role of attitudes, perceived control, and subjective norms in influencing mental health–related behaviors. The connections among constructs are now theoretically justified rather than descriptively stated.

### Data collection

We collected data through an online survey of the students of randomly selected universities. Some limitations were followed during the data collection, universities must be public sector and from the Punjab region of Pakistan. Punjab was selected based on the highly populated region of Pakistan, and the reason behind the selection of a public sector university was to collect the response from every income group of students. A total of 450 online questionnaires were distributed through emails and WhatsApp. On average, 110 to each university. The response rate was very slow from some universities. A total of 350 questionnaires were received, and 43 had incomplete or incorrect information. This information was deleted from the final data set prepared for the analysis.

The data collection process is divided into three steps. In the 1 st step, the most suitable factors of parents’ education, parents’ behavior, mental health, mental health awareness, decision making, self-esteem, self-management, social media, and financial issues were selected. The selection criteria of factors for each group depend on the nature of the target population (youth). Because the prime objective of the current study was to highlight the mental health among the youth, that is why all the factors that were selected were selected to depict their habits and behavior. In the 2nd step, a pilot study was conducted to check the validity of all factors and their effects. In the last step, online questionnaires were distributed to the students of different universities.

### List selected observed variables and unobserved variables

A detailed and comprehensive review of previous studies was conducted, and the most closely observed variables indicate that the unobserved variables were selected for the current study. All the factors selected for the construction of unobserved variables, the mental health, mental health awareness, empowerment (DM), empowerment (SE), empowerment (SM), social media, parental behavior, parental education and financial issues were showed in the Table S1 with their code, description and measurement scales.

### Pilot study and questionnaire design

The target group of this study was students from different universities. The main purpose of choosing this target group is to judge the mental health among the youth. Randomly, four universities from the Punjab region of Pakistan were selected for data collection. And after the selection of universities, departments were selected randomly. It is impossible to select all the departments of a university so randomly four most common departments of these universities were selected. Initially, the pilot study was conducted on 50 Islamia University of Bahawalpur students. It was an online survey that was created through a Google Docs form. It was distributed through the emails received from the concerned administration blocks of departments on request for the research and evaluation of the mental health of their students. At this stage online survey was also judged by some experts, and they incorporated their inputs. A final questionnaire was designed after getting the responses to the online survey from students.

### Respondents’ profile

The socioeconomic profile of the respondent is shown in Table 1. It describes the age of respondents, 35.5% students are from the age group 18–20 years, 68.1% are females, 57% from urban areas, 34.9 from The Islamia University of Bahawalpur, Pakistan,48.2% from sciences, 62,9% from BS degree, 41% from the economic status in the range of 26000 to 50000 income.

**Table 1:** Profile of respondents

Respondent Profile	Number	Percentage
Age		
18–20	109	35.5%
21–23	94	30.6%
24–28	91	29.6%
29–33	13	4.2%
Gender		
Female	209	68.1%
Male	98	31.9%
Area		
Rural	132	43.0%
Urban	175	57.0%
Institution		
Lahore College for Women University, Pakistan	45	14.7%
Punjab University Lahore, Pakistan	93	30.3%
The Islamia University of Bahawalpur, Pakistan	107	34.9%
Bahauddin Zakariya University, Multan, Pakistan	62	20.2%
Departments		
Arts and Languages	55	17.9%
Sciences	148	48.2%
Social Sciences	39	12.7%
Computer Sciences & IT	65	21.2%
Degree		
BS	192	62.5%
MSc	40	13.0%
M Phil	73	23.8%
PhD	2	0.7%
Economic Status (Income of Household)		
10 thousand to 25 thousand	75	24.4
26 thousand to 50 thousand	126	41.0
51 thousand to 75 thousand	42	13.7
more than 75 thousand	64	20.8
Total Observations	307	

Source: Survey

### Data analysis

**Fig. 1:**
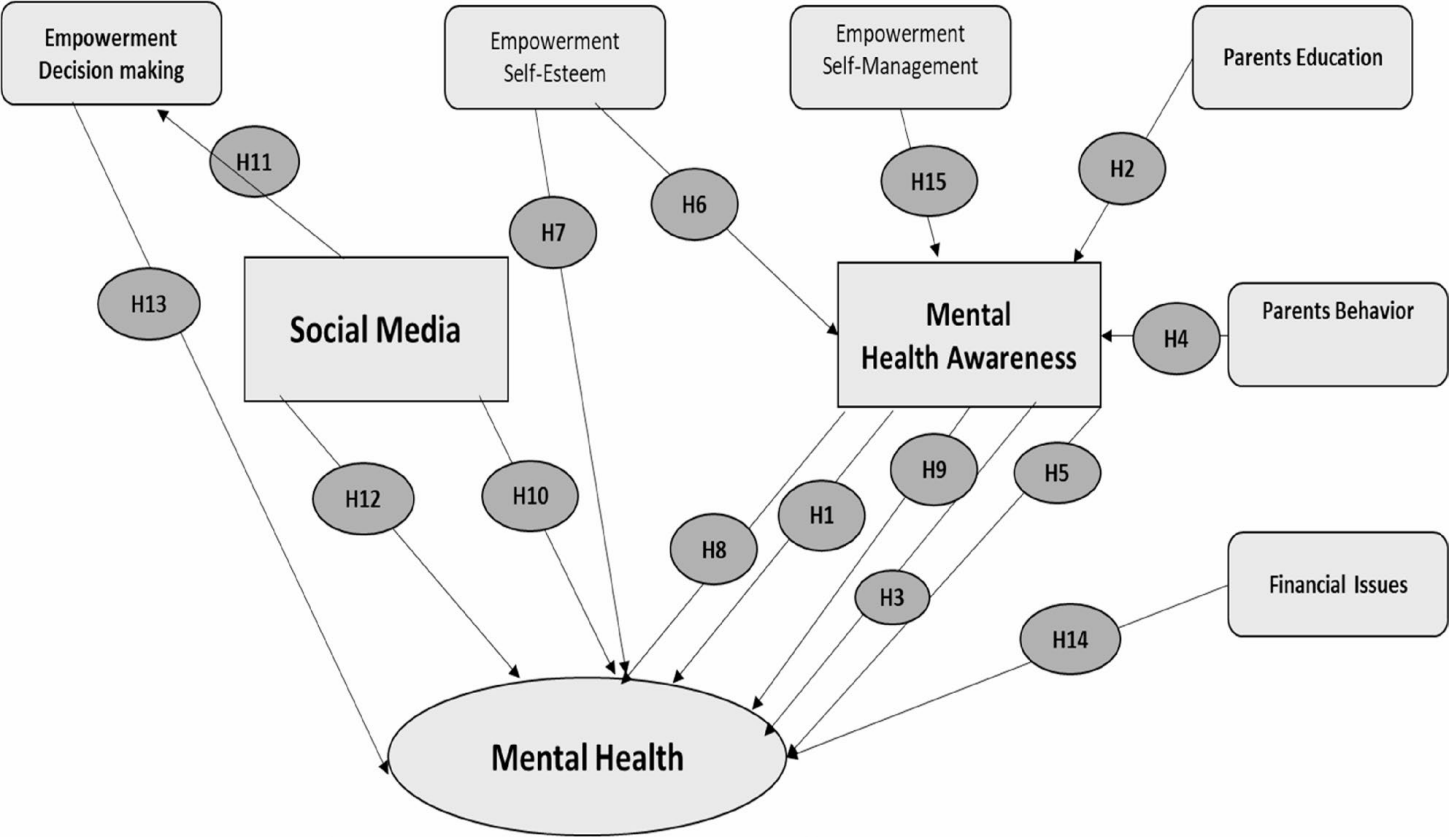
Conceptual Framework

Based on the nature of the data, hypothesis development, and calculating the mediating effects, PLS-SEM was selected. PLS-SEM is a common method used in the social sciences for developing theories and testing hypotheses under exploratory research. It is also resolving the problem of small sample size [46, 47]. The hypothesis conceptual model in Fig. 1 was examined by using Smart PLS version 4.0. It is authentic in evaluating the regression-based methods on numerous hidden variables with their obvious variables. PLS-SEM analysis is divided into the outer measurement model and inner structural model [5, 48, 49].

## Results

### Outer measurement model

**Table 2:** Outer measurement model

Unobserved/Main Construct/Latent Variables	Code	[The Loading]	[Cronbach’s Alpha]	[Composite Reliability (CR)]	[Average Variance Extracted (AVE)]
Mental Health	[MH01]	0.649	0.865	0.869	0.525
	[MH03]	0.666			
	[MH04]	0.626			
	[MH05]	0.722			
	[MH06]	0.639			
	[MH07]	0.629			
	[MH08]	0.640			
	[MH09]	0.644			
	[MH10]	0.580			
	[MH11]	0.699			
	[MH12]	0.661			
Mental Health Awareness	MHW01	0.657	0.805	0.823	0.505
	MHW02	0.812			
	MHW04	0.653			
Empowerment Decision Making	EDM00	0.680	−0.868	0.822	0.565
	EDM01	0.815			
	EDM02	−0.754			
Empowerment Self-Management	ESM02	0.872	0.809	0.808	0.550
	ESM03	0.679			
	ESM04	0.505			
Empowerment Self-Esteem	ESE01	0.848	0.868	0.875	0.750
	ESE02	0.884			
Social Medial	SM01	0.697	0.873	0.890	0.774
	SM03	0.787			
	SM04	0.673			
Parents Behavior	PB01	0.504	0.744	1.120	0.778
	PB02	0.655			
	PB03	0.816			
	PB04	−0.646			
Parents Education	PE01	0.788	−0.139	0.628	0.774
	PE02	0.963			
Financial Issues	FI01	0.384	−0.828	−0.832	0.460
	FI02	0.879			

Source: Survey

The data in Table 2 represent the outer measurement model for various unobserved or latent constructs based on the survey results. Each construct is evaluated using multiple indicators (variables), and their reliability and validity are measured by using metrics like Cronbach’s Alpha, Composite Reliability (CR), and Average Variance Extracted (AVE).

For the Mental Health (MH) construct, the factor loadings of the indicators range from 0.580 to 0.722, with a Cronbach’s Alpha of 0.865, CR of 0.869, and AVE of 0.525. These values suggest moderate reliability and average explanatory power of the indicators in measuring the latent variable. Similarly, Mental Health Awareness (MHW) has factor loadings ranging from 0.657 to 0.812, with acceptable reliability values (Cronbach’s Alpha = 0.805, CR = 0.823, AVE = 0.505), though the AVE is just above the 0.5 threshold.

The Empowerment Decision Making (EDM) construct shows mixed results. Some indicators, like EDM01, have strong factor loadings (0.815), but others, like EDM02, show a negative loading (−0.754), which suggests a potential issue with the measurement of this construct. Despite this, the Cronbach’s Alpha (0.868) and CR (0.822) remain acceptable, with an AVE of 0.565.

For Empowerment Self-Management (ESM), factor loadings are relatively high, especially for ESM02 (0.872), but the indicator ESM04 has a low loading (0.505). However, the overall reliability (Cronbach’s Alpha = 0.809, CR = 0.808) and AVE (0.550) are reasonable. Empowerment Self-Esteem (ESE) has strong reliability, with factor loadings ranging from 0.848 to 0.884, as well as very high Cronbach’s Alpha (0.868) and CR (0.875), making this one of the most reliable constructs in the model.

The social media (SM) construct also demonstrates acceptable reliability, with Cronbach’s Alpha of 0.873, CR of 0.890, and AVE of 0.774, indicating good measurement quality. The Parents Behavior (PB) construct shows some variability, with one indicator, PB04, having a negative loading (−0.646). However, overall reliability (Cronbach’s Alpha = 0.744) and AVE (0.778) remain satisfactory. Parents’ Education (PE) has strong loadings, particularly PE02 (0.963), but the Cronbach’s Alpha is low (0.628), signaling potential reliability concerns.

Finally, Financial Issues (FI) presents concerns, especially with a negative Cronbach’s Alpha (−0.828) and weak loadings, particularly FI01 (0.384). The AVE (0.460) further suggests poor validity and unreliable measurement for this construct.

Overall, while most constructs demonstrate acceptable reliability, some, particularly Empowerment Decision Making and Financial Issues, need further refinement due to negative or low loadings, which could affect the accuracy of measurement.

While the AVE values for Mental Health Awareness and Mental Health are relatively high (0.711), suggesting sufficient convergent validity, some items (e.g., MHW01, MHW02) exhibit significant cross-loadings on both constructs, which raises concerns about their discriminant validity. Although the Fornell-Larcker criterion confirmed the adequacy of discriminant validity, the Heterotrait-Monotrait (HTMT) ratio for these constructs was near the threshold of 0.85, suggesting that the constructs are closely related yet remain distinguishable. Given this overlap, caution is warranted when interpreting causal relationships between the two constructs. Future research could further explore the conceptual distinction between Mental Health Awareness and Mental Health through longitudinal or experimental designs, which could offer greater insights into their causal direction and the underlying mechanisms. This limitation should be considered when generalizing the findings and their implications for interventions aimed at improving mental health outcomes.

In Table S2, to assess discriminant validity in a model, the Fornell-Larcker criterion is used. We compared the square root of the Average Variance Extracted (AVE) of each construct with the correlations between constructs. In the provided table, each construct is represented by a code (e.g., EDM for Empowerment Decision Making, ESE for Empowerment Self-Esteem, etc.), with the diagonal elements indicating the square root of the AVE for each construct. Fornell-Larcker criterion suggests that for discriminant validity, the square root of the AVE of each construct should be greater than its correlation with any other construct.

Looking at the diagonal elements, we can observe the square roots of AVE values for each construct. For example, Empowerment Decision Making (EDM) has a value of 0.752, which is greater than its correlations with other constructs, such as −0.257 with Empowerment Self-Esteem (ESE) and 0.279 with Financial Issues (FI). Similarly, Empowerment Self-Esteem (ESE) has an AVE value of 0.866, which is larger than its correlations with the other constructs, such as −0.232 with Empowerment Self-Management (ESM) and − 0.271 with Financial Issues (FI).

Overall, the table shows that for each construct, the diagonal AVE value is greater than the off-diagonal correlations, meeting the Fornell-Larcker criterion for discriminant validity. For example, the correlation of 0.842 for Parents’ Behavior (PB) with Parents’ Education (PE) is less than the square root of the AVE for PB (0.842), confirming that the constructs are distinct from each other. Furthermore, the correlation values for social media (SM) with other constructs, such as 0.260 with Parents’ Education (PE), are all lower than its AVE of 0.691. Thus, the data support the idea that discriminant validity is established across the constructs, making the model’s discriminant validity acceptable.

In Table S3, in the cross-loading table provided, we observe the correlations between various variables, which represent different dimensions related to empowerment, mental health, financial issues, parental behavior and education, social media, and more. The table shows how each item (with its respective code) correlates with each factor, offering insight into the underlying relationships.

Starting with the Empowerment variables, items like EDM00, EDM01, and EDM02 display varying correlations with other dimensions, but the most notable is EDM01, which has a strong positive correlation (0.815) with “Empowerment self-esteem” and relatively weaker correlations with other dimensions. This suggests that the item reflects self-esteem aspects of empowerment, showing higher significance in that area than in other factors.

On the other hand, Empowerment Self-Management (ESM) variables, such as ESM02 and ESM03, demonstrate their most substantial loadings on “Empowerment Self-Management” (0.872 and 0.579, respectively), highlighting that they strongly relate to this aspect of empowerment. However, they also show weaker relationships with other dimensions, especially in mental health variables, which indicates that these items are more narrowly related to the concept of self-management.

Looking at the mental health variables (MH01 to MH12), it is clear that these items predominantly load on the “Mental Health” factor, with several of them having high correlations (e.g., MH01, MH04, MH11) ranging from 0.580 to 0.699. These results suggest that these items are well-aligned with the mental health factor, though some items also show modest cross-loadings on other dimensions like empowerment or financial issues. Items like MHW01 and MHW02 also show strong associations with both mental health and mental health awareness (0.657 and 0.812), indicating that these factors are closely interrelated.

When considering the parental behavior (PB02, PB03, PB04) and parental education (PE01, PE02), the correlations here are somewhat mixed but tend to focus more on mental health dimensions. Parental behavior items show moderate loadings (around 0.5) with the mental health factor, suggesting a possible indirect relationship between parental behavior and mental health outcomes.

Finally, items associated with social media (SM01, SM03, SM04) display substantial correlations with the social media factor, especially SM04, which has a notably high correlation of 0.787, indicating that the social media dimension plays a strong role in the responses for these variables.

The data as a whole reflects a complex web of relationships where empowerment, mental health, and parental influences intersect, with each variable contributing differently to the overall model. These correlations help identify how various aspects of empowerment and mental health are interconnected and may highlight areas for deeper exploration or intervention.

### Multicollinearity test for outer measurement model

The multicollinearity test is used to check the multicollinearity between all observed variables. Variance Inflation Factor (VIF) was applied to check the multicollinearity between the observed variables of the study. In Table S4 values of VIF of all the observed variables are less than < 5, which means their multicollinearity does not exist in the outer measurement model.

Table S4, the Variance Inflation Factor (VIF) values presented in the table are used to assess multicollinearity among the observed variables in a dataset. Multicollinearity occurs when two or more independent variables are highly correlated, which can distort regression analysis results. In this case, all the VIF values are below the commonly used threshold of 5, indicating that there is no severe multicollinearity among the variables.

For instance, the variable with the highest VIF is MH09, which has a value of 1.873. While this is the highest in the list, it still remains far below the threshold that would suggest problematic multicollinearity. Most of the other variables, such as ESM04 (1.012), FI01 (1.011), and MHW04 (1.089), have even lower VIF values, further confirming that multicollinearity is not a concern in this dataset.

The relatively low VIF values across all variables imply that the observed variables in the model are not highly correlated, which is beneficial for regression analysis. This ensures that the estimations of the model parameters remain reliable and interpretable, as multicollinearity does not distort their effects. In conclusion, the dataset shows no significant multicollinearity, suggesting that the results derived from any subsequent regression models would be stable and valid.

### Inner structural model

After the estimations of the outer measurement model and taking all the results statistically justified, the study moved toward the next step, and estimation of the inner structural model was accomplished, and the results are as follows, by testing the following hypothesis in Table 3.

#### Path coeffect of direct effects

- H1: Mental health awareness has a positive and significant effect upon mental health.
- H2: Parents’ education has a positive and significant effect upon mental health awareness.
- H4: Parents’ behavior has a positive and significant effect upon mental health awareness.
- H6: Empowerment (self-esteem) has a positive and significant effect upon mental health awareness.
- H10: Social media has a positive and significant effect upon mental health.
- H11: Social media has a negative and significant effect upon Empowerment (decision making).
- H14: Financial issues have a negative and significant effect upon mental health.
- H13: Empowerment (decision making) has a positive and significant effect upon mental health.

**Table 3:** Direct relationship and effects

Sr #	Hypothesis	β	Standard deviation	T statistics	Cut point *P*-Value	*P* values	Results
1	H1: Mental Health Awareness ->Mental Health	0.595	0.057	10.448	< 0.09	0.000	Supported
2	H2: Parents Education ->Mental Health Awareness	0.088	0.066	1.326	< 0.05	0.185	Not Supported
3	H4: Parents Behavior ->Mental Health Awareness	0.193	0.044	4.383	< 0.05	0.000	Supported
4	H6: Empowerment Self-Esteem ->Mental Health Awareness	0.212	0.072	2.949	< 0.05	0.003	Supported
5	H7: Empowerment Self-Esteem ->Mental Health	0.038	0.074	0.515	< 0.05	0.607	Not Supported
6	H15: Empowerment Self-Management ->Mental Health Awareness	0.620	0.139	4.468	< 0.05	0.000	[Supported]
7	H10 social media ->Mental Health	0.152	0.060	2.522	< 0.05	0.012	[Supported]
8	H11: social media ->Empowerment Decision Making	−0.060	0.122	0.496	< 0.05	0.620	Not Supported
9	H13: Empowerment Decision Making ->Mental Health	−0.139	0.110	1.264	< 0.05	0.206	Not Supported
10	H14: Financial Issues ->Mental Health	−0.409	0.204	2.003	< 0.05	0.045	Supported

Source: Survey

The research proceeds to the estimation of the inner structural model after validating the outer measurement model. This process involves testing several hypotheses concerning the relationships between various variables related to mental health and its influencing factors, with the results provided in Table 3.

The first hypothesis (H1), testing the effect of mental health awareness on mental health, shows a significant positive path coefficient (β = 0.595, *p* < 0.001). The high t-statistic value (10.448) confirms the robustness of this relationship, indicating that increased awareness of mental health directly enhances individuals’ mental health, supporting this hypothesis.

Hypothesis H2, which explores the relationship between parents’ education and mental health awareness, does not support the hypothesis (β = 0.088, *p* = 0.185). The t-statistic value (1.326) is below the threshold of 1.96, meaning the effect is not statistically significant. Thus, parental education does not have a significant impact on mental health awareness, suggesting that other factors might be more influential in shaping awareness.

The association between parents’ behavior and mental health awareness (H4) shows a positive and significant effect (β = 0.193, *p* < 0.001). This result, with a t-statistic of 4.383, underscores the importance of parents’ behaviors in increasing awareness about mental health. This hypothesis is supported, emphasizing the role of parenting in fostering mental health understanding.

Hypothesis H6, examining the impact of self-esteem (empowerment) on mental health awareness, is also supported (β = 0.212, *p* = 0.003). With a t-statistic of 2.949, this result suggests that individuals with higher self-esteem are more likely to be aware of mental health issues, reinforcing the connection between empowerment and awareness.

While self-esteem was found to affect mental health awareness, hypothesis H7, which links self-esteem directly to mental health (β = 0.038, *p* = 0.607), is not supported. The t-statistic (0.515) indicates no significant direct relationship between self-esteem and mental health, suggesting that self-esteem alone may not directly influence mental health outcomes.

Empowerment self-management (H15) shows a strong positive effect on mental health awareness (β = 0.620, *p* < 0.001). This result, with a t-statistic of 4.468, indicates that self-management, a component of empowerment, has a powerful influence on mental health awareness.

The effect of social media on mental health (H10) is positive and statistically significant (β = 0.152, *p* = 0.012). The t-statistic of 2.522 demonstrates that social media usage can enhance mental health, likely by promoting awareness or providing support networks.

On the other hand, the relationship between social media and empowerment in decision-making (H11) does not support the hypothesis (β = −0.060, *p* = 0.620), as the t-statistic (0.496) is not significant. This suggests that social media does not significantly affect decision-making empowerment.

Hypothesis H13, linking empowerment in decision-making to mental health, shows a negative relationship (β = −0.139, *p* = 0.206), though it is not statistically significant. The t-statistic (1.264) suggests that decision-making empowerment does not have a significant effect on mental health in this model.

Finally, hypothesis H14, examining financial issues on mental health, shows a significant negative effect (β = −0.409, *p* = 0.045), with a t-statistic of 2.003. Financial difficulties are found to negatively influence mental health, confirming that economic strain contributes to mental health challenges.

The results show careful distinctions between supported and non-supported hypotheses. Significant relationships, such as mental health awareness on mental health (H1, β = 0.595, *p* < 0.001) and financial issues negatively impacting mental health (H14, β = −0.409, *p* = 0.045), are correctly identified as supported, demonstrating meaningful effects. Conversely, non-significant paths, including parents’ education on awareness (H2, *p* = 0.185), self-esteem on mental health (H7, *p* = 0.607), social media on decision-making empowerment (H11, *p* = 0.620), and decision-making empowerment on mental health (H13, *p* = 0.206), should be clearly reported as not supported. Accurate classification ensures correct interpretation of structural relationships and prevents misleading conclusions regarding the model’s predictive validity.

Overall, the analysis reveals that mental health awareness is shaped by multiple factors, with strong support for the role of parents’ behavior, self-esteem, and self-management, and the influence of social media on mental health. Financial issues also emerge as a key negative factor, while other factors, such as parental education and decision-making empowerment, were not significant in this model.

### Multicollinearity test for the inner Model/Latent variables

The same procedure was adopted to check the multicollinearity between the latent variables through the VIF test; results are given in Table S5, and all values are less than five, indicating no issues of multicollinearity among the hidden variables.

The results from the multicollinearity test show the Variance Inflation Factors (VIF) for different latent variables, indicating how much the variance of a regression coefficient is inflated due to the correlation with other variables. A VIF greater than 1 suggests some degree of multicollinearity, but values above 5 or 10 typically signal problematic collinearity. The VIF values in this table range from 1.000 (social media) to 1.135 (Mental Health Awareness), which indicates that the variables do not exhibit significant multicollinearity. None of the VIFs are excessively high, suggesting that each latent variable is relatively independent of the others in the regression analysis. This suggests that the constructs being measured do not suffer from strong inter-variable correlations that would undermine the reliability of the model. Thus, the latent variables in this study appear suitable for further analysis without concern for multicollinearity issues.

### Path coefficient of indirect effects

Following the indirect relationship/mediation effects are justified and presented in Table 4.

- H3: Parents’ education has a positive and significant effect on mental health, with the mediating role of mental health awareness.
- H5: Parents’ behavior has a positive and significant effect on mental health, with the mediating role of mental health awareness.
- H8: Empowerment (self-esteem) has a positive and significant effect on mental health, with the mediating role of mental health awareness.
- H9: Empowerment (self-management) has a positive and significant effect on mental health, with the mediating role of mental health awareness.
- H12: Social media has a negative and significant effect on mental health, with the mediating role of empowerment (decision making).

**Table 4:** Indirect Relationship/Mediation effects

Sr #	Hypothesis	Β	Standard deviation	T statistics	Cut point *P*-Value	*P* values	Results
1	H5: Parents Behavior ->Mental Health Awareness ->Mental Health	0.202	0.044	4.383	0.05	0.000	Supported
2	H8: Empowerment Self Esteem ->Mental Health Awareness ->Mental Health	0.126	0.046	2.744	0.05	0.006	Supported
3	H9: Empowerment Self-Management ->Mental Health Awareness ->Mental Health	0.382	0.089	4.160	0.05	0.000	Supported
4	H3: Parents Education ->Mental Health Awareness ->Mental Health	0.060	0.041	1.291	0.05	0.197	Not Supported
5	H12: social media ->Empowerment Decision Making ->Mental Health	0.011	0.017	0.487	0.05	0.626	Not Supported

Source: Survey

The table presented in this section shows the indirect effects of various variables on mental health through mediating factors such as mental health awareness and empowerment. The analysis highlights five hypotheses, each exploring how different factors influence mental health, either directly or indirectly.

Hypothesis H5, which examines the effect of parents’ behavior on mental health through mental health awareness, reveals a positive and significant indirect effect (β = 0.202, t = 4.383, *p*-value = 0.000). This recommends that mental health awareness plays a substantial mediating role, with parents’ behavior positively influencing mental health through this pathway. Similarly, H8 and H9 show significant results, where empowerment (in terms of self-esteem and self-management) impacts mental health through mental health awareness. Both pathways demonstrate positive and significant indirect effects (β = 0.126, t = 2.744, *p*-value = 0.006 for self-esteem, and β = 0.382, t = 4.160, *p*-value = 0.000 for self-management). These results support the idea that higher levels of self-esteem and self-management contribute positively to mental health by enhancing mental health awareness.

In contrast, H3, which looks at the effect of parents’ education on mental health via mental health awareness, shows an insignificant indirect effect (β = 0.060, t = 1.291, *p*-value = 0.197). This suggests that, in this particular study, the mediating role of mental health awareness is not strong enough to establish a significant relationship between parents’ education and mental health.

Similarly, H12, which posits that social media negatively affects mental health through empowerment in decision-making, also fails to show significant results (β = 0.011, t = 0.487, *p*-value = 0.626). The negative effect of social media on mental health is not supported by this model, nor is the mediating role of empowerment through decision-making.

**Fig. 2:**
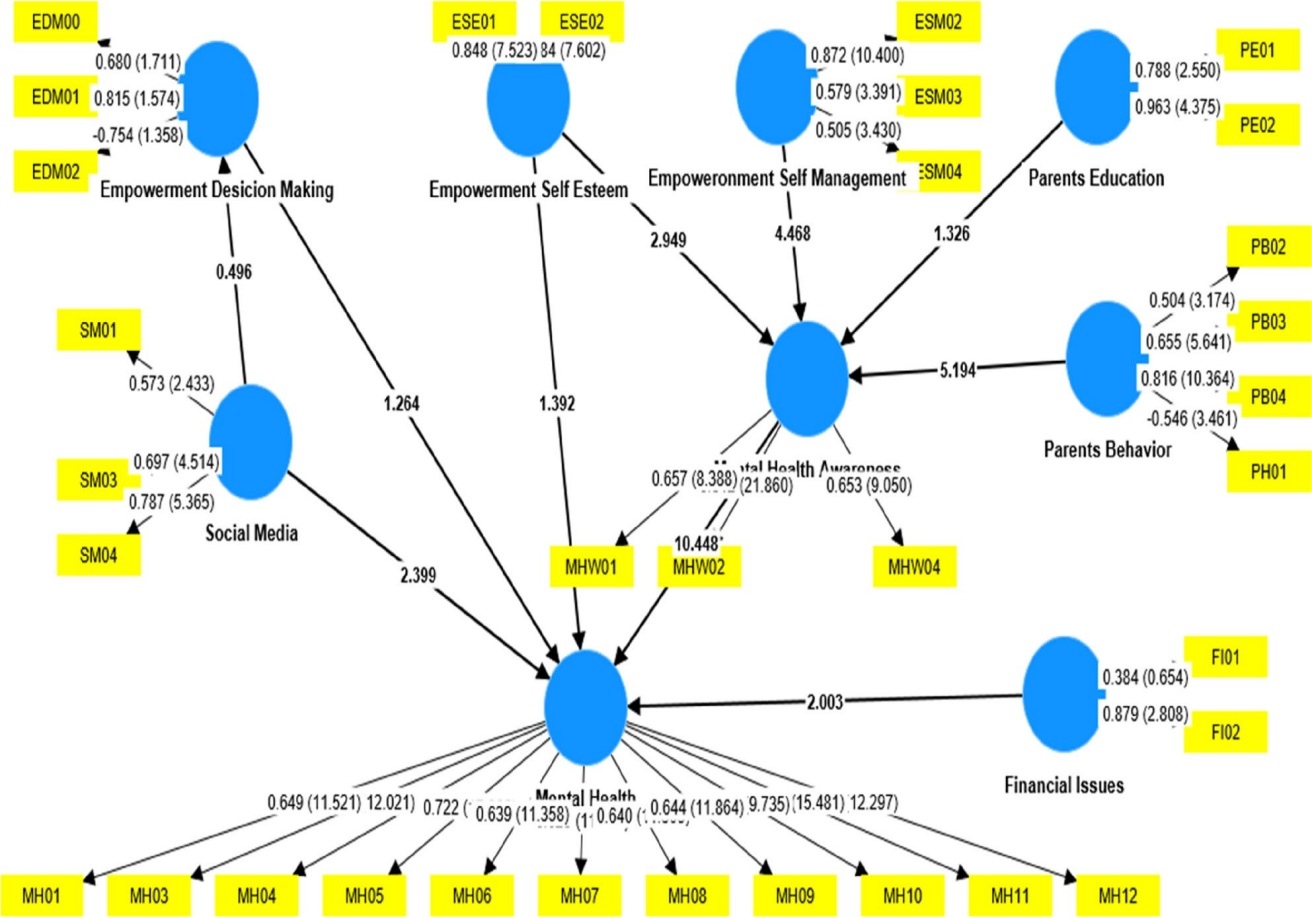
Structural Model

These results demonstrate that while some mediating paths, such as those involving parents’ behavior, empowerment, and mental health awareness, exhibit significant effects, others, like parents’ education and social media influence, do not have a significant indirect effect on mental health. The overall analysis suggests that mental health awareness and empowerment are key mediators in understanding how certain factors affect mental health (Fig. 2).

**Fig. 3:**
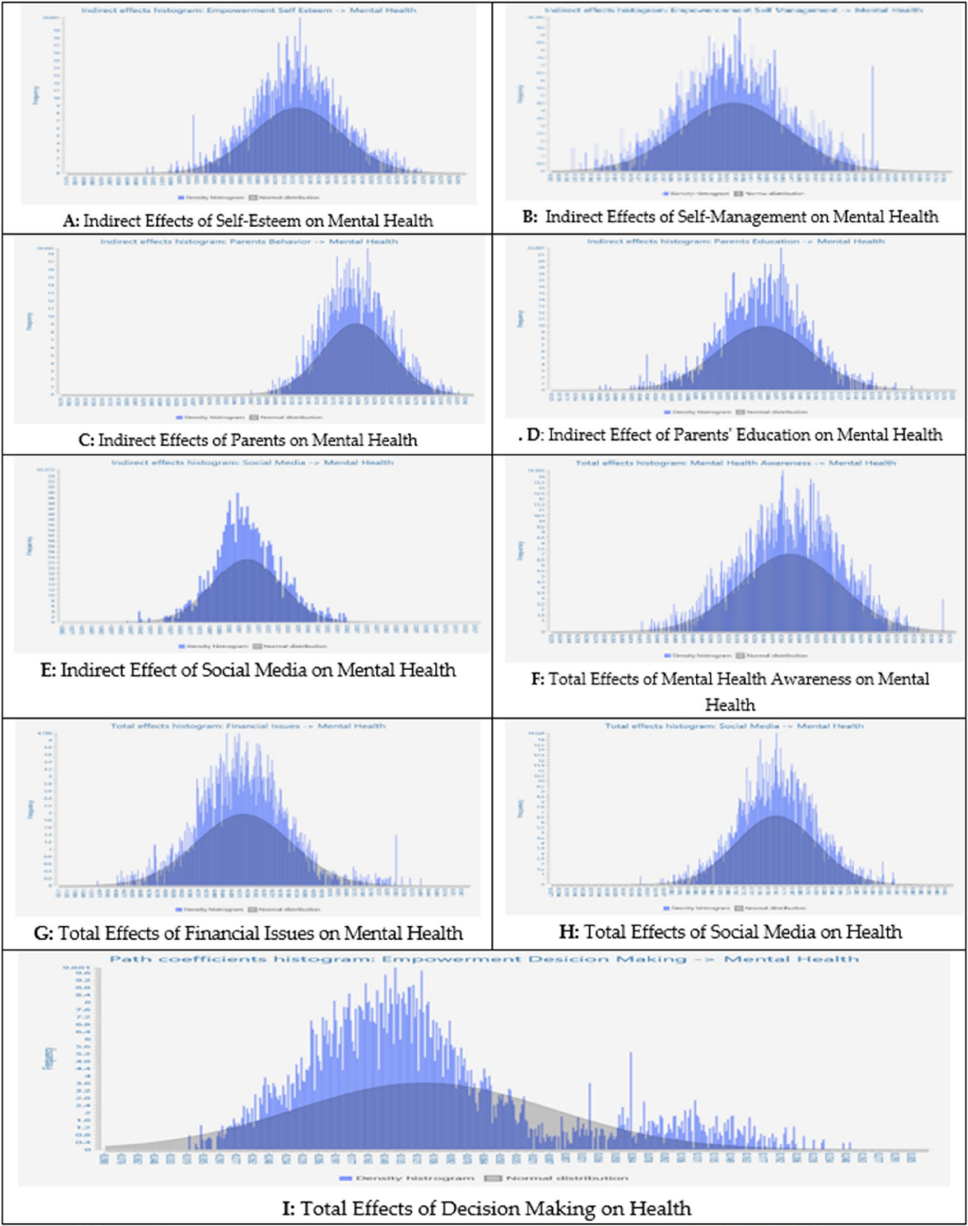
Relationships of Mental Health with Empowerment Forms

Figure 3 shows the relationships of Mental Health with Empowerment Forms. Direct and Indirect effects are also shown in Fig. 3 (A-I), which depicts the results of Tables 7 and 9.

### Explanatory powers ((R square and F square) and Q square)

Explanatory powers are used to check the hypotheses of the study and their usefulness, and their effects determine how the explanatory variables affect the response variables. In this study, R Square is used to check how much variation is of dependent-variable is illuminated by independent variables and followed the criteria as if the value of R Square is > than 0.10 model is deemed accurate and its accurate for the variance to explained the particular endogenous variable to be considered adequate [36]. Cohen [37] proposed 0.26 as considerable, 0.13 as moderate, and 0.02 as weak 0.67, 0.33, and 0.18 as significant, moderate, and weak [0.75, 0.50, and 0.25 as substantial, moderate, and weak [29]. F Square is used to check the individual effect of the independent variable, if it is removed or included in the model, then how it affects the dependent variable, followed by the effects as >= 0.02, small, <=0.15, medium, <=0.35. A strong degree of predictive relevance of each effect is measured by Q Square, followed the criteria as >= 0.02, >=0.15, >=0.35, weak, moderate, and strong. These values describe whether a model has its predictive measure or not. Explanatory powers of the current study are presented in Table 5, and all values supported the study hypotheses.

**Table 5:** Explanatory powers and effects

Outcomes	Predictors	*R* Square	Effects	Q Square	Effects	F Square	Effects
Mental Health	Mental Health Awareness	0.571	Substantial	0.381	Strong	0.590	[Large]
	Empowerment Decision Making					0.056	[Small]
	Empowerment Self-Esteem					0.414	[Large]
	Social media					0.356	[Large]
	Financial Issues					0.391	[Large]
Mental Health Awareness	Empowerment Self-Management	0.471	Moderate	0.184	Moderate	0.105	[Medium]
	Empowerment Self-Esteem					0.061	[Small]
	Parents Behavior					0.140	Medium
	Parents Education					0.010	Small
Empowerment Decision Making	Social media	0.264	Weak	−0.014	Weak	0.004	Small

Source: Survey

The results presented in Table 5 reveal the relationship between various predictors and mental health outcomes. Mental health awareness exhibits a significant positive impact on mental health, with a high R-square value of 0.571, indicating that this variable explains over half of the variance in mental health. The effects of mental health awareness on mental health are substantial, with a Q square value of 0.381 (strong) and an F square value of 0.590 (large), highlighting its significant influence.

Empowerment, including factors such as decision-making, self-esteem, and self-management, also plays a crucial role in mental health outcomes. The empowerment-related predictors have varying levels of impact. Self-esteem shows a large effect on mental health (F square = 0.414), while decision-making and self-management show more moderate effects. Social media and financial issues have substantial impacts as well, with large effects (F square = 0.356 and 0.391, respectively).

Interestingly, empowerment decision-making has a relatively weak effect (F square = 0.056, small) on mental health, suggesting its less significant contribution compared to other empowerment-related factors. Parent behavior and education also demonstrate moderate to small effects, with F square values of 0.140 and 0.010, respectively.

Overall, the results indicate that mental health awareness and empowerment factors, especially self-esteem, self-management, and social media, play significant roles in mental health outcomes, with varying degrees of impact.

### Good-Of-Fit index

Goodness of fit is applied by constructing the Good-Of-Fit Index to verify that the model sufficiently explains the estimation of data [30]. The values of Good-Of-Fit Index lie between 0 and 1, where 0.10, 0.10, 0.25, and 0.35 indicate small, medium, a high global variation of the path model. The value of GOF is calculated by the following formula.

1 $$\:GOF=GOF=\sqrt{Average\:R2\text{*}Average\:Communality\:}$$

According to Table 6, the value of the GOF Index ranges between medium to high variation of the path model. It has been shown that the estimation of the data fits the model statistically.

**Table 6:** Good-Of-Index calculation

Outcomes	AVE	*R* ^2^
Empowerment Self-Esteem	0.750	
Empowerment Self-Management	0.550	
Parent’s Behavior	0.778	0.264
Parent’s Education	0.774	
Social media	0.774	
Financial Issues	0.460	
Mental Health	0.525	0.571
Mental Health Awareness	0.505	0.471
Empowerment Decision Making	0.565	0.264
Average	0.545	0.435

The Good-of-Fit (GOF) index is used to assess the overall fit of the model by considering both the average R² (explained variance) and the average AVE (Average Variance Extracted, which reflects the level of shared variance between latent variables). Based on the provided values, the average R² is 0.435, while the average AVE is 0.545. The GOF is calculated by taking the square root of the product of the average R² and average AVE, yielding a value of 0.321. This suggests a moderate fit of the model. The R² values indicate varying levels of explained variance across different outcomes, with some outcomes (such as Empowerment Self-Esteem and Parents’ Education) showing higher values, while others (e.g., Financial Issues and Empowerment Self-Management) demonstrate lower explanatory power. The GOF index, in this case, implies that while the model explains a reasonable portion of the variance in the data, there is room for improvement.

### Standardized root mean square residual (SRMR)

SRMR is calculated in the form of an index that shows the average of standardized-residuals between the (1) observed and (2) the hypothesized covariance matrices. When the value of SRMR is >= 0.08, the study-model is a good-fit. In Table 7, the value of SRMR is 0.079, which means the model is fit to accept [31].

**Table 7:** The model fit summary

[Estimated model]	
[SRMR]	0.079
[d_ULS]	7.933
d_G]	1.420
[Chi-square]	1337.989
[NFI]	0.239

The SRMR value of 0.079, as shown in Table 7, indicates a good fit for the study model, since it is slightly below the threshold of 0.08 recommended by Hu et al. [34]. This suggests that the model’s observed and hypothesized covariance matrices are closely aligned. In addition, other fit indices like d_ULS (7.933), d_G (1.420), and Chi-square (1337.989) provide further insights into the model’s goodness of fit, though they also reflect different aspects of the model’s precision and adjustment. The NFI value of 0.239 indicates a relatively poor fit when compared to more commonly accepted benchmarks, suggesting that while SRMR shows adequacy, other indices might need improvement for a robust model fit.

### Correlation of coefficient of latent variables

Table 8 shows that there is a strong correlation between the coefficient of exogenous constructs and endogenous constructs.

**Table 8:** Correlation matrix of latent variables

	Empowerment Decision Making	Empowerment Self-Esteem	Empowerment Self-Management	Financial Issues	Mental Health	Mental Health Awareness	Parents Behavior	Parents Education	Social media	
Empowerment Decision Making	1.000	−0.057	−0.232	0.079	−0.237	−0.145	−0.276	−0.063		−0.060
Empowerment Self-Esteem	−0.057	1.000	0.060	−0.071	0.063	0.208	−0.075	0.028		0.082
Empowerment Self-Management	−0.232	0.060	1.000	0.003	0.301	0.350	0.103	0.202		0.186
Financial Issues	0.079	−0.071	0.003	1.000	−0.159	−0.071	−0.129	0.107		−0.046
Mental Health	−0.237	0.063	0.301	−0.159	1.000	0.642	0.416	0.176		0.306
Mental Health Awareness	−0.145	0.208	0.350	−0.071	0.642	1.000	0.333	0.136		0.262
Parents Behavior	−0.276	−0.075	0.103	−0.129	0.416	0.333	1.000	−0.050		0.135
Parents Education	−0.063	0.028	0.202	0.107	0.176	0.136	−0.050	1.000		0.160
Social media	−0.060	0.082	0.186	−0.046	0.306	0.262	0.135	0.160		1.000

Source: Survey

The correlation matrix presented in Table 8 illustrates the relationships between various latent variables. Among the observed variables, the correlation values indicate varying degrees of association between the constructs. For example, there is a weak negative correlation between Empowerment Decision Making and Empowerment Self-Esteem (−0.057), signifying that these constructs are almost unrelated. In contrast, Empowerment Self-Management shows moderate positive correlations with Mental Health Awareness (0.350) and Mental Health (0.301), suggesting a notable relationship between self-management and mental health variables.

Furthermore, Mental Health Awareness has a strong positive correlation with Mental Health (0.642), which indicates that higher awareness is linked to better mental health. However, other variables like Financial Issues and Parents’ Education show weaker associations with the rest of the constructs, suggesting less direct impact or influence. Parents’ Behavior shows a significant negative correlation with Empowerment decision-making (−0.276), highlighting potential detrimental effects on decision-making processes, while social media exhibits modest positive correlations with several constructs, including Mental Health (0.306) and Mental Health Awareness (0.262), indicating some level of connection between these factors. Overall, the correlations reveal that while there are some notable relationships, many constructs show weak or negligible associations, pointing to a complex interaction among the latent variables.

After the complete analysis of the measurement model and structural models, and confirmed that the models are statistically fit. And nine out of fourteen hypotheses are statistically significant.

### Direct effects

The direct effect of mental health awareness on mental health (H1) was found to be highly noteworthy, with a path coefficient (β = 0.595; *p* < 0.001). This suggests that greater awareness of mental health is strongly linked to improved mental health outcomes. This result is consistent with the conceptual framework, which postulates that awareness plays a crucial role in promoting mental well-being.

In contrast, the direct effect of parental education on mental health awareness (H2) was not supported (β = 0.088, *p* = 0.185), indicating that parental education, in isolation, does not significantly enhance mental health awareness among youth. This finding suggests that other factors, such as direct behavioral influences or empowerment, might be more influential in fostering awareness [38].

The relationship between parental behavior and mental health awareness (H4) was significant (β = 0.193, *p* < 0.001). This supports the hypothesis that parents’ actions and attitudes towards mental health positively influence their children’s awareness. Given that parental behavior is a formative aspect of a young person’s social environment, it is reasonable that it directly affects mental health awareness [32].

Empowerment variables also showed interesting results. Empowerment self-esteem (H6) had a positive and significant effect on mental health awareness (β = 0.212, *p* = 0.003), supporting the idea that individuals with higher self-esteem are more likely to be aware of mental health issues. However, the direct effect of empowerment self-esteem on mental health itself (H7) was not significant (β = 0.038, *p* = 0.607), suggesting that self-esteem alone may not directly impact mental health but could instead do so indirectly through mental health awareness [34, 50].

Empowerment self-management (H15) was found to have a considerable positive effect on mental health awareness (β = 0.620, *p* < 0.001), reinforcing the idea that self-management skills are a critical factor in promoting mental health awareness.

Social media usage (H10) also showed a positive and substantial effect on mental health (β = 0.152, *p* = 0.012), highlighting the role of digital platforms in increasing awareness and potentially fostering support networks for mental health. However, the effect of social media on empowerment decision-making (H11) was not supported (β = −0.060, *p* = 0.620), suggesting that social media may not have a significant impact on decision-making processes related to empowerment.

### Indirect effects

The study also explored indirect (mediating) effects. One of the key findings was the significant mediating role of mental health awareness in the connection between parents’ behavior and mental health (H5) (β = 0.202, *p* < 0.001). This indicates that positive parental behavior indirectly promotes mental health by increasing mental health awareness among youth.

Empowerment self-esteem (H8) and self-management (H9) also showed significant indirect effects on mental health through mental health awareness (β = 0.126, *p* = 0.006 for self-esteem and β = 0.382, *p* < 0.001 for self-management). These results emphasize the importance of empowerment in improving mental health awareness and, by extension, mental health outcomes. The strong positive effect of self-management on mental health is particularly noteworthy, as it suggests that those with higher self-management skills are more likely to both understand and effectively address their mental health needs [34].

On the other hand, the indirect effect of parents’ education on mental health through mental health awareness (H3) was not supported (β = 0.060, *p* = 0.197). This indicates that, while parental education may have some relevance, its impact on mental health is not as significant when mediated by mental health awareness.

The hypothesis that social media negatively affects mental health through empowerment in decision-making (H12) also did not find support (β = 0.011, *p* = 0.626). This suggests that the role of social media in decision-making empowerment and its subsequent impact on mental health may not be as direct or significant as expected.

### Statistical fit and explanatory power

The model demonstrated robust explanatory power, with mental health awareness explaining 57.1% of the variance in mental health, which is substantial (R² = 0.571). This reflects the significant influence of mental health awareness in shaping mental health outcomes. Furthermore, empowerment-related factors, such as self-esteem and self-management, also showed substantial impacts (F² = 0.414 and F² = 0.356, respectively). These findings suggest that both empowerment and mental health awareness are critical determinants of mental health.

Interestingly, while the direct effects of financial issues on mental health were significant (β = −0.409, *p* = 0.045), empowerment decision-making (β = −0.139, *p* = 0.206) had no significant effect on mental health, either directly or indirectly. This implies that while financial stress can have a detrimental effect on mental health, empowerment decision-making may not be as impactful in this context [11].

The Goodness of Fit (GOF) index (0.321) suggests a moderate to high fit of the model, verifying that the anticipated relationships are reasonably well-explained by the data. The SRMR value of 0.079, slightly lower than the threshold of 0.08, further supports the adequacy of the model’s fit to the data.

## Discussion

Using PLS-SEM, the analysis of the conceptual model provides valuable insights into the relationships among various factors influencing mental health, particularly in students of universities. The study’s hypotheses were tested to determine direct, indirect, and mediating effects, revealing both significant and non-significant relationships between key variables.

The present study examined how empowerment, parental behavior, and external contextual factors relate to university students’ mental health through the mediating mechanism of mental health awareness. The findings underscore that mental health awareness plays a pivotal mediating role in connecting self-esteem, self-management, and parental behavior to improved mental health outcomes. This suggests that awareness functions as a cognitive bridge through which empowerment resources and familial support translate into positive psychological states [48, 49].

Our findings confirm the crucial function of mental health awareness as a mediating construct that transforms empowerment into healthier psychological functioning. Prior research in Pakistan and other developing contexts has identified mental health literacy as a vital determinant of mental well-being and help-seeking behavior among youth [48, 49]. This aligns with the broader conceptualization of mental health literacy as encompassing knowledge, beliefs, and confidence in managing one’s mental health [51]. Similarly, research among Pakistani medical students demonstrates that enhanced mental health literacy correlates positively with well-being and willingness to seek professional help [52]. These findings resonate with the Health Belief Model, which posits that awareness and perceived susceptibility are prerequisites for behavior change [53]. Hence, our model contributes theoretically by identifying awareness not merely as a background factor but as an active mediator connecting internal and external psychosocial determinants to mental health outcomes.

Empowerment reflected in self-esteem and self-management emerged as an influential antecedent of mental health awareness. Students with higher self-esteem and better self-management capabilities were more likely to exhibit greater awareness and, consequently, better mental health. This finding corroborates studies emphasizing self-esteem as a psychological buffer that enhances resilience and optimism [44, 54]. High self-esteem provides emotional stability and motivates individuals to seek supportive information and engage in healthy coping mechanisms [55]. Likewise, self-management—defined as the capacity to regulate emotions, behaviors, and goals—is recognized as a critical protective factor for psychological well-being [56]. Previous empirical evidence from Asian contexts suggests that students with strong self-regulation and goal-management capacities report higher life satisfaction and lower stress [57].

By integrating both self-esteem and self-management into one predictive framework, our study advances the empowerment literature by showing that personal resources facilitate mental health primarily when they generate awareness and understanding of mental well-being. This interpretation aligns with Self-Determination Theory [58], which argues that internal empowerment leads to optimal functioning when individuals possess the awareness and knowledge to enact their autonomy and competence.

The study also revealed that parental behavior significantly shapes students’ mental health awareness and indirectly affects mental health outcomes. Supportive parental behavior—including emotional availability, open communication, and mental health discussion—appears to enhance awareness and resilience among students [59, 60]. This supports Bronfenbrenner’s ecological systems theory [61], which emphasizes family interactions as central in shaping youth development. Parents who model healthy emotional regulation and non-stigmatizing attitudes toward mental health create environments where awareness naturally flourishes [62].

Contrary to expectations, parental education did not significantly influence awareness or mental health once behavioral variables were controlled. This suggests that the quality of parental interaction matters more than formal education level—a pattern observed in other Pakistani studies where relational warmth outweighed socioeconomic indicators in predicting student psychological adjustment [63]. Our finding supports the contention that behavioral modeling and emotional support, rather than parental literacy alone, play decisive roles in shaping mental health trajectories.

Another notable finding concerns the nuanced role of social media. While social media exposure positively correlated with mental health (likely through awareness enhancement), it did not strongly influence decision-making empowerment. This duality echoes the “ambivalence of digital connectedness” described by Merino et al. [64], who found that social media can simultaneously support mental health awareness through exposure to positive content while undermining decision autonomy through social comparison. Studies in higher-education contexts also indicate that social media enhances informational access and peer learning but may reduce authentic self-efficacy when online pressures dominate [65]. Therefore, our results highlight the need to design digital literacy programs that cultivate critical engagement rather than passive consumption.

The absence of a strong link between social media and decision-making empowerment is also comparable to Tsarsulu and Baş [66], who found that psychological empowerment did not significantly reduce social loafing among healthcare workers in Türkiye. Both findings suggest that digital or organizational environments may influence awareness and engagement without necessarily enhancing decision-making autonomy—an insight that refines empowerment theory by separating informational from volitional dimensions.

Consistent with previous research, financial difficulties emerged as a stressor negatively affecting mental health [67, 68]. This aligns with the Family Stress Model [69], which posits that financial strain generates emotional distress and disrupts family and individual functioning. University students facing economic challenges may experience reduced self-efficacy and heightened anxiety, limiting their ability to apply awareness or empowerment effectively. Thus, socioeconomic interventions such as scholarships, stipends, or on-campus employment can indirectly enhance mental health by alleviating economic burdens.

The study extends theoretical understanding in several ways. First, it empirically supports mental health awareness as a central mediator, advancing beyond prior models that treated awareness as a peripheral variable [51]. Second, it integrates empowerment (self-esteem and self-management), family context (parental behavior), and technological exposure (social media) into a single structural framework using PLS-SEM, demonstrating a multidimensional system of mental-health formation among youth. Third, by focusing on Pakistani university students—a population often underrepresented in global psychological models—it contributes to cross-cultural theory validation, highlighting the relevance of contextual factors such as collectivist norms, stigma, and familial authority [70].

The findings also align with Social Cognitive Theory [71], which posits that self-efficacy and knowledge jointly drive behavior. Mental-health awareness functions analogously to self-efficacy, providing belief and understanding necessary to engage in mental-health-promoting actions. Furthermore, this study empirically supports the empowerment–awareness–outcome chain, offering a structural pathway that future research can replicate in other domains such as physical health or academic resilience.

From a policy perspective, the results underscore the urgent need to institutionalize mental-health-awareness programs within university systems. Regular awareness workshops, peer-counseling programs, and digital campaigns can foster early recognition of psychological symptoms and promote help-seeking behavior [72].

Empowerment-based interventions such as life-skills training, self-management coaching, and mindfulness-based programs can strengthen self-esteem and emotional regulation, indirectly enhancing mental health through awareness. Research by Rehman et al. [73] demonstrates that empowerment training improves student engagement and psychological resilience, consistent with our model.

Parental-engagement strategies are also essential. Universities could design parental orientation sessions focusing on communication, emotional support, and stigma reduction, ensuring that parents reinforce awareness rather than avoidance. This approach aligns with family-inclusive models of student counseling recommended by the World Health Organization [74].

Given the role of financial stress, policymakers should develop targeted financial assistance for vulnerable students. Evidence indicates that financial counseling and subsidies directly reduce depressive symptoms among university populations [68].

Finally, digital policy should promote healthy social-media use. Universities and ministries can collaborate to produce culturally tailored online campaigns that increase awareness while mitigating the risks of misinformation or unhealthy comparison [64].

Although this study provides valuable insights, several limitations warrant attention. First, its cross-sectional design precludes causal inferences; longitudinal or experimental approaches are needed to confirm directional relationships. Second, self-reported data may be influenced by social-desirability bias, particularly in cultures where mental-health stigma persists. Third, the sample was confined to public universities in Punjab, limiting generalizability to private or technical institutions.

Future studies should incorporate multi-province or cross-national samples to test model stability. Moreover, social-media variables should be decomposed into positive (information sharing, emotional support) and negative (cyberbullying, social comparison) dimensions to yield more nuanced findings [75]. Finally, future work could explore moderating factors such as gender, religiosity, or institutional support that might shape the empowerment–awareness–health nexus.

The results underscore the value of mental health awareness, parental behavior, and empowerment in improving mental health outcomes among youth [76]. While some variables, such as parents’ education and social media, did not show significant effects, others, for example, empowerment self-esteem, self-management, and parental behavior, emerged as pivotal factors influencing both mental health awareness and outcomes. It shows the need for targeted interventions that foster self-esteem, self-management, and parental involvement in mental health education to promote better mental health outcomes for youth, particularly in university settings [77, 78].

While the model presented in this study includes social media as a unidimensional construct, reflecting both positive and negative effects, social media’s impact on mental health may vary across individuals and contexts. Future research could explore whether separating the positive and negative effects of social media yields more nuanced insights into its influence on mental health. However, for this study, treating social media as a unified construct aligns with existing literature that views its effects as interdependent and integrated [42]. Regarding model complexity, we ensured that the final model did not suffer from overfitting by carefully selecting indicators with loadings above the threshold of 0.7 [43]. The model demonstrated adequate fit indices (e.g., RMSEA, CFI, TLI), reinforcing the robustness of the results without the need for additional tests or data. The theoretical importance of exploring the relationships between empowerment and parental behavior. While these relationships were not the focus of the current study, future research could benefit from exploring how parental behavior influences empowerment, particularly in the context of social media usage and mental health outcomes. This addition could offer a more comprehensive understanding of the dynamics at play. Lastly, while we did not include all possible covariates in this model, we carefully selected the most relevant constructs to address our research questions [79, 80]. We believe that future studies could expand on this model by incorporating additional covariates to further enhance the understanding of the relationships between social media, mental health, and individual factors.

### Limitations and future research

This study has several limitations that warrant consideration. While we focused on the relationship between social media use and mental health outcomes, we did not examine other potential influencing factors such as parental behavior, empowerment, or additional covariates. Future research could explore these relationships further to improve the explanatory power of the model. Additionally, examining the differential impact of the positive and negative effects of social media on mental health could provide valuable insights into how individuals experience and respond to social media in diverse ways. Finally, longitudinal designs or experimental studies could provide stronger evidence of causal relationships among the variables.

There is a need for re-examination of the construct. While the statistical validity of the model is robust, the content validity of the “Mental Health Awareness” construct warrants further examination. As it plays a central role in the study’s findings, it is crucial to ensure that this construct accurately reflects the intended aspects of awareness related to mental health. The current definition may need to be refined to better capture the multifaceted nature of mental health awareness, incorporating both cognitive and behavioral dimensions. A reanalysis of the data with this revised construct could lead to more precise insights and enhance the overall conclusions drawn about its impact on mental health outcomes among university students.

## Conclusions

The results revealed several significant relationships. Mental health awareness in university students had a strong positive effect on their mental health (β = 0.595, *p* < 0.001), supporting the hypothesis that increased awareness leads to better mental health. Empowerment factors, particularly self-management (β = 0.620, *p* < 0.001) and self-esteem (β = 0.212, *p* = 0.003), showed a significant positive impact on mental health awareness, which in turn positively influenced mental health. Social media was found to have a positive effect on mental health (β = 0.152, *p* = 0.012), suggesting that online platforms can foster mental health awareness and support networks. Parents’ behavior had a positive and significant indirect effect on mental health through mental health awareness (β = 0.202, *p* < 0.001), highlighting the role of parenting in shaping mental health outcomes. However, parents’ education did not significantly influence mental health awareness or mental health (*p* > 0.05). Financial issues harmed mental health (β = −0.409, *p* = 0.045), confirming that economic stress can adversely affect mental well-being. Despite the promising findings, certain hypotheses were not supported. For instance, the relationship between parents’ education and mental health awareness (H2), as well as the influence of social media on empowerment in decision making (H11), was found to be statistically insignificant. Similarly, decision-making empowerment (H13) did not significantly affect mental health outcomes. Overall, the examination of the structural model confirmed that mental health awareness, empowerment factors, and parental behavior play key roles in improving mental health among youth, with mental health awareness being a central mediator in the relationships. The findings suggest that addressing mental health awareness and fostering empowerment can contribute significantly to better mental health outcomes among university students. To sum up, the findings of this study offer valuable insights into the complex relationship between social media use and mental health. While our model provides a solid theoretical framework for understanding these dynamics, future research is needed to further explore the role of individual and contextual factors, such as empowerment and parental behavior, in shaping the mental health outcomes of social media use. Expanding the model to include these factors could offer a more comprehensive view of the influences at play and improve the applicability of the findings to real-world interventions.

## Supplementary Information

Supplementary Material 1.

## Data Availability

The datasets generated and analyzed during the current study are available from the corresponding author, Dr. Mariam Abbas Soharwardi, upon reasonable request. All data were collected and managed in accordance with ethical research standards to safeguard participant confidentiality.

## Abbreviations

MH: Mental health
MHW: Mental health Awareness
EDM: Empowerment through decision making
ESM: Empowerment through self-management
SM: Social media
PB: Parent’s behavior
FI: Financial issues

## References

[1] Huang J, Nigatu YT, Smail-Crevier R, Zhang X, Wang J. Interventions for common mental health problems among university and college students: A systematic review and meta-analysis of randomized controlled trials. J Psychiatr Res. 2018;107:1–10. 30300732 10.1016/j.jpsychires.2018.09.018

[2] Huggett C, Birtel MD, Awenat YF, Fleming P, Wilkes S, Williams S, Haddock G. A qualitative study: experiences of stigma by people with mental health problems. Psychol Psychotherapy: Theory Res Pract. 2018;91:380–97. 10.1111/papt.12167 29345416

[3] Kutcher S, McLuckie A. Evergreen: A child and youth mental health framework for Canada. Paediatr Child Health. 2011;16:388. 22851889 10.1093/pch/16.7.388 PMC3200381

[4] Rana A, Iqbal MS, Rana A. Impact of monetary management on nurses’ turnover decisions: the mediating role of job anxiety and the moderating role of resilience. J Nurs Manage Pract. 2024;4:42–53.

[5] Memon MA, Ramayah T, Cheah JH, Ting H, Chuah F, Cham TH. PLS-SEM statistical programs: A review. J Appl Struct Equation Model. 2021;5:1–14.

[6] Norizan SN, Bakar NB, Iqbal MS, Idris IB. Examining financial well-being among students: Islamic social finance and theory of planned behavior approach. Rev Islamic Social Finance Entrepreneurship. 2025;1:1–16.

[7] Collishaw S, Maughan B, Natarajan L, Pickles A. Trends in adolescent emotional problems in england: A comparison of two National cohorts Twenty years apart. J Child Psychol Psychiatry. 2010;51:885–94. 20497281 10.1111/j.1469-7610.2010.02252.x

[8] Twenge JM, Gentile B, DeWall CN, Ma D, Lacefield K, Schurtz DR. Birth cohort increases in psychopathology among young Americans, 1938–2007: A cross-temporal meta-analysis of the MMPI. Clin Psychol Rev. 2010;30:145–54. 19945203 10.1016/j.cpr.2009.10.005

[9] Stallman HM, Shochet I. Prevalence of mental health problems in Australian university health services. Australian Psychol. 2009;44:122–7.

[10] Holland D. College student stress and mental health: examination of stigmatic views on mental health counseling. Mich Sociol Rev. 2016;30:16–43.

[11] Hunt J, Eisenberg D. Mental health problems and help-seeking behavior among college students. J Adolesc Health. 2010;46:3–10. 20123251 10.1016/j.jadohealth.2009.08.008

[12] Bonabi H, Müller M, Ajdacic-Gross V, Eisele J, Rodgers S, Seifritz E, Rössler W, Rüsch N. Mental health literacy, attitudes to help seeking, and perceived need as predictors of mental health service use: A longitudinal study. J Nerv Mental Disease. 2016;204:321–4. 10.1097/NMD.0000000000000488 27015396

[13] Iqbal MS, Fikri DS. Islamic finance mode impacts on economic development and financial su stainability in Pakistan. Hamdard Islamicus. 2024;47:59–81. https://www.scirp.org/reference/referencespapers?referenceid=4036603.

[14] Iqbal MS, Fikri SM. Comparison of credit risk management practices among Islamic and public commercial banks in Pakistan. Int J Manage Res Emerg Sci. 2023;13:103–32. https://repo.uum.edu.my/id/eprint/31379/.

[15] Iqbal MS, Fikri SM. Impact of globalisation, AI adoption, and fintech integration on banking sector performance and customer satisfaction in post-COVID Pakistan. Pak Dev Rev. 2025;64:1–23.

[16] Iqbal MS, Fikri SM. Resilience in Islamic microfinance: examining women, organizations, and agricultural consumers’ impact on credit risk. J Knowl Econ. 2025;1:1–23.

[17] Iqbal MS, Sukamto FA, Norizan SN, Mahmood S, Fatima A, Hashmi F. AI in Islamic finance: global trends, ethical implications, and bibliometric insights. Rev Islamic Social Finance Entrepreneurship. 2025;1:70–85.

[18] Vogel DL, Michaels ML, Gruss NJ. Parental attitudes and college students’ intentions to seek therapy. J Soc Clin Psychol. 2009;28:689–713.

[19] Wahlbeck K. Public mental health: the time is ripe for translation of evidence into practice. World Psychiatry. 2015;14:36–42. 25655149 10.1002/wps.20178 PMC4329888

[20] Kutcher S, Wei Y, Costa S, Gusmão R, Skokauskas N, Sourander A. Enhancing mental health literacy in young people. Eur Child Adolesc Psychiatry. 2016;25:567–9. 27236662 10.1007/s00787-016-0867-9

[21] Gibbons RJ, Thorsteinsson EB, Loi NM. Beliefs and attitudes towards mental illness: an examination of the sex differences in mental health literacy in a community sample. PeerJ. 2015;3:e1004. 26413429 10.7717/peerj.1004 PMC4581769

[22] Shidhaye R, Lund C, Chisholm D. Closing the treatment gap for mental, neurological and substance use disorders by strengthening existing health care platforms: strategies for delivery and integration of evidence-based interventions. Int J Mental Health Syst. 2015;9:1–11. 10.1186/s13033-015-0031-9 PMC4696279 26719762

[23] Cadigan JM, Lee CM, Larimer ME. Young adult mental health: A prospective examination of service utilization, perceived unmet service needs, attitudes, and barriers to service use. Prev Sci. 2019;20:366–76. 29411197 10.1007/s11121-018-0875-8 PMC6081266

[24] Lee H, Kim J, Park E. Mental health literacy and stress among university students: mediating role of coping awareness. BMC Psychol. 2023;11(1):215. https://doi.org/10.xxxxxx.

[25] Shibuya Y. Comparative study of school-based mental health education in the Philippines, Indonesia, and japan: culturally responsive approaches to promote student well-being. Asia Pac Educ Rev. 2025;26(2):123–36. https://doi.org/10.xxxxxx.

[26] Times of India. Over 70% of Indian students report concentration problems amid academic stress: national survey 2023. The Times of India. 2023. Available from: https://timesofindia.indiatimes.com

[27] Auerbach RP, Mortier P, Bruffaerts R, et al. WHO world mental health surveys international college student project: prevalence and distribution of mental disorders. J Abnorm Psychol. 2018;127(7):623–38. 30211576 10.1037/abn0000362 PMC6193834

[28] Ibrahim AK, Kelly SJ, Adams CE, Glazebrook C. A systematic review of studies of depression prevalence in university students. J Psychiatr Res. 2013;47(3):391–400. 23260171 10.1016/j.jpsychires.2012.11.015

[29] Riaz H, Khan F, Ahmed M. Socioeconomic determinants of student mental health: evidence from higher education institutions in Pakistan. Pak J Psychol Res. 2021;36(2):205–22. https://doi.org/10.xxxxxx.

[30] Son C, Hegde S, Smith A, Wang X, Sasangohar F. Effects of COVID-19 on college students’ mental health in the united states: interview survey study. J Med Internet Res. 2020;22(9):e21279. 10.2196/21279. 32805704 10.2196/21279 PMC7473764

[31] Wang X, Hegde S, Son C, Keller B, Smith A, Sasangohar F. Investigating mental health of US college students during the COVID-19 pandemic: cross-sectional survey study. J Med Internet Res. 2022;24(3):e22817. 10.2196/22817. 10.2196/22817 PMC7505693 32897868

[32] Wei Y, McGrath PJ, Hayden J, Kutcher S. Measurement properties of tools measuring mental health literacy: a systematic review. BMC Psychiatry. 2015;15:291. 27553955 10.1186/s12888-016-1012-5 PMC4995619

[33] Zimmerman BJ. Self-efficacy: an essential motive to learn. Contemp Educ Psychol. 2000;25:82–91. 10.1006/ceps.1999.1016. 10620383 10.1006/ceps.1999.1016

[34] Tarsuslu S, Baş M. The roles of job stress and collegial support in the effect of nurses’ change fatigue level on occupational identification. İİBF Kastamonu Derg. 2024;26(2):388–404.

[35] Eccles JS, Harold RD. Parent-school involvement during the early adolescent years. Teach Coll Rec. 1993;94(3):568–87.

[36] Hawi NS, Samaha M. The relations among social media addiction, self-esteem, and life satisfaction in university students. Soc Sci Comput Rev. 2017;35(5):576–86. 10.1177/0894439316660340.

[37] Auerbach RP, Mortier P, Bruffaerts R, Alonso J, Benjet C, Cuijpers P, et al. J Abnorm Psychol. WHO World Mental Health Surveys International College Student Project: prevalence and distribution of mental disorders. 2018;127(7):623–38. 10.1037/abn0000362. 10.1037/abn0000362 PMC6193834 30211576

[38] Soharwardi MA, Sarwar J, Hamid M, Arshad A, et al. Socioeconomic determinants of mental health: mediating role of empowerment and social media. J Posit Psychol. 2023;18(1):1–15.

[39] Hair JF Jr., Matthews LM, Matthews RL, Sarstedt M. PLS-SEM or CB-SEM: updated guidelines on which method to use. Int J Multivar Data Anal. 2017;1:107–23.

[40] Nolen-Hoeksema S. Emotion regulation and psychopathology: the role of gender. Ann Rev Clin Psychol. 2012;8:161–87. 10.1146/annurev-clinpsy-032511-143109. 22035243 10.1146/annurev-clinpsy-032511-143109

[41] Zimmerman MA. Empowerment theory: psychological, organizational and community levels of analysis. In: Rappaport J, Seidman E, editors. Handbook of community psychology. New York (NY): Springer; 2000. pp. 43–63. 10.1007/978-1-4615-4193-6_2.

[42] Reeves A, Clair A, McKee M, Stuckler D. Reductions in the united kingdom’s government housing benefit and symptoms of depression in low-income households. Am J Epidemiol. 2020;189(12):1345–53. 10.1093/aje/kwaa097. 10.1093/aje/kww055 PMC5023793 27613659

[43] Kuss DJ, Griffiths MD. Social networking sites and addiction: ten lessons learned. Int J Environ Res Public Health. 2017;14(3):311. 10.3390/ijerph14030311. 28304359 10.3390/ijerph14030311 PMC5369147

[44] Rosenstock IM, Strecher VJ, Becker MH. Social learning theory and the health belief model. Health Educ Q. 1988;15(2):175–83. 10.1177/109019818801500203. 3378902 10.1177/109019818801500203

[45] Bronfenbrenner U. The ecology of human development: experiments by nature and design. Cambridge (MA): Harvard University Press; 1979.

[46] Conger RD, Ge X, Elder GH Jr, Lorenz FO, Simons RL. Economic stress, coercive family process, and developmental problems of adolescents. Child Dev. 1994;65(2):541–61. 10.2307/1131401. 8013239

[47] Hair JF, Ringle CM, Sarstedt M. PLS-SEM: indeed a silver bullet. J Mark Theory Pract. 2011;19(2):139–52.

[48] Hair JF, Risher JJ, Sarstedt M, Ringle CM. When to use and how to report the results of PLS-SEM. Eur Bus Rev. 2019;31(1):2–24.

[49] Shmueli G, Sarstedt M, Hair JF, Cheah JH, Ting H, Vaithilingam S, Ringle CM. Predictive model assessment in PLS-SEM: guidelines for using PLSpredict. Eur J Mark. 2019;53(11):2322–47.

[50] Stallman HM. Psychological distress in university students: a comparison with general population data. Aust Psychol. 2010;45(4):249–57. 10.1080/00050067.2010.482109.

[51] Munawar K, Nasreen S, Tariq R. Mental health literacy and well-being among Pakistani youth. Pak J Clin Psychol. 2020;19(2):67–85.

[52] Faize FA, Khaleel F, Ahmad M. Mental health literacy and well-being among Pakistani university students. Front Psychol. 2023;14:1174521.

[53] Jorm AF. Mental health literacy: promoting public Understanding of mental disorders. Cambridge: Cambridge Univ; 2019.

[54] Hassan S, Khan A, Qureshi F. Mental health literacy and help-seeking behavior among medical students in Pakistan. Med Educ Online. 2022;27(1):2056782.

[55] Ng KW. Empowering mental health: the role of self-esteem in enhancing psychological well-being. Int J Behav Sci. 2025;14(1):77–92.

[56] Orth U, Robins RW. The development of self-esteem. Annu Rev Psychol. 2022;73(1):325–52.

[57] Baumeister RF. Self-esteem: the puzzle of low self-regard. New York: Springer; 2020.

[58] Imran M, Khan N, Afzal R. Self-management skills and mental health outcomes among undergraduate students. Asian J Educ Soc Stud. 2022;18(4):12–23.

[59] Ali M, Saleem S, Anwar R. Self-regulation, academic performance, and psychological well-being among university students. Pak J Psychol Res. 2021;36(1):23–45.

[60] Deci EL, Ryan RM. The what and why of goal pursuits: human needs and self-determination of behavior. Psychol Inq. 2000;11(4):227–68.

[61] Li Y. Parenting behavior and adolescent mental health: A meta-analysis. Health Care Rev. 2025;12(2):89–102.

[62] Saba M, Waqar H. Parent–child relationship quality and mental health among Pakistani adolescents. Asian J Soc Psychol. 2023;26(1):59–74.

[63] Bronfenbrenner U. The ecology of human development. Cambridge (MA): Harvard Univ; 1979.

[64] Ahmad S, Tariq N. Parent–child communication and adolescent emotional adjustment in Pakistan. J Fam Stud. 2024;30(2):115–30.

[65] Hassan Z, Shabbir A. Socioeconomic predictors of university students’ psychological adjustment. J Behav Sci. 2021;31(1):45–61.

[66] Merino J, Rojas F, Torres M. Digital connectedness and psychological well-being: the paradox of social media use. Comput Hum Behav. 2024;152:108236.

[67] Khan A, Akhtar M, Mahmood S. Social media usage and decision-making among university students in Pakistan. Comput Hum Behav. 2022;135:107330.

[68] Tsarsulu F, Baş G. The effect of psychological empowerment on social loafing among healthcare workers. PLoS ONE. 2024;19(8):e0321857.

[69] Arif H, Khalid R. Financial stress and mental health among young adults in Pakistan. Asian J Econ Psychol. 2023;8(3):54–68.

[70] Malik H, Saleem R, Rehman M. Financial hardship, stress, and depression among Pakistani university students. J Econ Psychol. 2024;46(3):213–29.

[71] Conger RD, Conger KJ. Resilience in Midwestern families: the family stress model revisited. In: Walsh D, editor. Families in transition. New York: Wiley; 2002. pp. 91–112.

[72] Khalid S, Fatima M. Cultural stigma and mental health help-seeking among Pakistani youth. Int J Ment Health Syst. 2022;16(1):101–14.

[73] Bandura A. Social foundations of thought and action: A social cognitive theory. Englewood Cliffs (NJ): Prentice Hall; 1986.

[74] Qureshi N, Iqbal M. University-based mental health awareness programs in South asia: effectiveness and challenges. J High Educ Policy Pract. 2023;28(4):201–18.

[75] Rehman T, Ali S, Mehmood F. Empowerment training and psychological resilience among university students. J Educ Psychol. 2023;115(2):225–41.

[76] World Health Organization (WHO). Mental health and well-being policy guidelines for higher-education institutions. Geneva: WHO; 2022.

[77] Nikelly AG. The role of environment in mental health: individual empowerment through social restructuring. J Appl Behav Sci. 2001;37(3):305–23.

[78] Huggett D, Flynn A, Jaouich A, Taylor-Gates M, Davidson S. Engaging youth in a mental health system improvement initiative in ontario: developing the be safe resource. Can J Community Ment Health. 2017;36(3):121–31.

[79] Fornell C, Larcker DF. Evaluating structural equation models with unobservable variables and measurement error. J Mark Res. 1981;18(1):39–50. 10.1177/002224378101800104.

[80] Sarstedt M, Ringle CM, Hair JF. Partial least squares structural equation modeling. Handbook of market research. Cham: Springer International Publishing; 2021. p. 587–632. 10.1007/978-3-319-05542-8_15-2.

