# Supplementary material for: Mental health and empowerment of university students: mediating role of mental health awareness
Source: BMC Psychol. 2025 Dec 4;14:18. doi: 10.1186/s40359-025-03726-5 (PMC12781656; doi:10.1186/s40359-025-03726-5)
Supplement: Supplementary file 1 — Supplementary Material 1. [file 40359_2025_3726_MOESM1_ESM.docx]

**Supplementary Material, Table S1:** Measurement Scales

| SR# | Self-Reported Questions | Description | Code | Measurement Scale |
| --- | --- | --- | --- | --- |
| 1 | I had trouble keeping my mind on what I was doing. | Mental Health  Indicates the symptoms of mental health judged with the feelings and observations of the respondent. And indicates from poor health to good health | MH01 | Every Day, all the time = 1  Twice a Week=2  Once a Week =3  Never=4 |
| 2 | I felt sad for no reason |  | MH03 |  |
| 3 | I could not get going to do good |  | MH04 |  |
| 4 | I felt like a bad person. |  | MH05 |  |
| 5 | I lost interest in my usual activities |  | MH06 |  |
| 6 | I felt like I was moving too slowly. |  | MH07 |  |
| 7 | I felt fidgety. |  | MH08 |  |
| 8 | I wished I were dead. |  | MH09 |  |
| 9 | I wanted to hurt myself. |  | MH10 |  |
| 10 | I was tired to happy all the time. |  | MH11 |  |
| 11 | I did not like myself. |  | MH12 |  |
| 12 | I can find the trouble getting to sleep | Mental Health Awareness  It is observed through the daily based actions or reactions of the respondents.  It also indicates negative feelings towards positive feelings | MHW01 | Always=1  Most of The Time =2  About half the time=3  Sometime=3  Never=4 |
| 13 | I know why I could not focus on the important things |  | MHW02 |  |
| 14 | I feel bored, cooped up, or antsy due to the environment of my class |  | MHW04 |  |
| 15 | Who decided about your study? | Empowerment  Decision Making  Measured through the daily based decisions of the respondent. This variable scales from low to high level empowerment | EDM00 | Some other=1  Relatives=2  Parents=3  Self-=4 |
| 16 | Whose choice is included in the University Selection |  | EDM01 |  |
| 17 | Who was choosing your subject? |  | EDM02 |  |
| 18 | Is your choice is included in the selection of the Supervisor? | Empowerment  Self-Management  Measured through the daily base level of satisfaction helps in the self-management of respondents. This variable scales from low to high level empowerment | ESM02 | No=0  Yes= 1 |
| 19 | Are you satisfied with your university's examination system? |  | ESM03 |  |
| 20 | Are you satisfied with your university's transport system? |  | ESM04 |  |
| 21 | Do you feel any humiliation in your class due to your family background? | Empowerment  Self-Esteem  Measure through the daily base experiences of respondents. This variable scales from high to low empowerment | ESE01 | Never=1  Sometime=2  ~ half the time=3  Most of The Time =4  Always=5 |
| 22 | My result in my class did not match my expectations |  | ESE02 |  |
| 23 | How often did you: text, email, or use the Internet while driving a motorized vehicle within the past 12 months? | Social Medial  Selected the social media that is most commonly used among the students for entertainment.  This variable scales from less use toward more use. | SM01 | Never=1  Sometime=2  ~Half the time=3  Most of The Time =4  Always=5 |
| 24 | How much time do you use the internet for Facebook? |  | SM03 |  |
| 24 | How much time do you use the internet for any other social media like TikTok or Snapchat? |  | SM04 |  |
| 25 | Are your parents living happily? | Parents Behavior  It indicates the parents’ behavior of with each other and with the respondent. This variable scales from poor to good behavior. | PB01 | Never=1  Always=1  ~Half the time = 2  Most of The Time =3  Sometimes=4  Never=5 |
| 26 | Are your parents fighting with each other? |  | PB02 |  |
| 27 | How often did a parent insult you at home? |  | PB03 |  |
| 28 | How often do your parents kick, pinch, or slap you at home |  | PB04 |  |
| 29 | Mother Education | Parents Education | PE01 | 5^th^ =1,  8^th^ =2  10^th^ =3  Higher=4 |
| 30 | Father Education |  | PE02 |  |
| 31 | Is your family facing any financial issues regarding university dues? | Financial Issues  These are the most common issues due to which students face tensions and depression, which ultimately affect their mental health | FI01 | No=0  Yes=1 |
| 32 | Are you doing any job to pay University fees? |  | FI02 |  |

Source: Survey

**Supplementary Material, Table S2:** Fornell-Larcker Criterion

| Code | EDM | ESE | ESM | FI | MH | MHW | PB | PE | SM |
| --- | --- | --- | --- | --- | --- | --- | --- | --- | --- |
| Empowerment Decision Making | **0.752** |  |  |  |  |  |  |  |  |
| Empowerment Self-Esteem | -0.257 | **0.866** |  |  |  |  |  |  |  |
| Empowerment Self-Management | -0.232 | 0.260 | **0.771** |  |  |  |  |  |  |
| Financial Issues | 0.279 | -0.271 | 0.003 | **0.778** |  |  |  |  |  |
| Mental Health | -0.237 | 0.263 | 0.301 | -0.159 | **0.652** |  |  |  |  |
| Mental Health Awareness | -0.145 | 0.208 | 0.350 | -0.071 | 0.642 | **0.711** |  |  |  |
| Parents Behavior | -0.276 | -0.275 | 0.103 | -0.129 | 0.416 | 0.333 | **0.842** |  |  |
| Parents Education | -0.063 | 0.028 | 0.202 | 0.107 | 0.276 | 0.136 | -0.250 | **0.880** |  |
| Social Media | -0.060 | 0.082 | 0.186 | -0.046 | 0.306 | 0.262 | 0.335 | 0.260 | **0.691** |

Source: Survey

**Supplementary Material, Table S3:** Cross-Loading

| Code | Empowerment Decision  Making | Empowerment  Self Esteem | Empowerment  Self- Management | Financial Issues | Mental Health | Mental Health Awareness | Parents Behavior | Parents Education | Social Media |
| --- | --- | --- | --- | --- | --- | --- | --- | --- | --- |
| EDM00 | 0.680 | -0.101 | -0.189 | 0.117 | -0.143 | -0.088 | -0.205 | -0.010 | -0.080 |
| EDM01 | 0.815 | -0.030 | -0.186 | 0.092 | -0.189 | -0.062 | -0.278 | -0.019 | 0.002 |
| EDM02 | -0.754 | 0.011 | 0.153 | 0.014 | 0.198 | 0.167 | 0.148 | 0.100 | 0.061 |
| ESE01 | -0.134 | 0.848 | 0.013 | -0.075 | 0.037 | 0.172 | 0.019 | -0.007 | 0.118 |
| ESE02 | 0.025 | 0.884 | 0.085 | -0.050 | 0.069 | 0.187 | -0.139 | 0.052 | 0.029 |
| ESM02 | -0.015 | 0.043 | 0.872 | 0.034 | 0.241 | 0.308 | 0.012 | 0.198 | 0.122 |
| ESM03 | -0.108 | 0.065 | 0.579 | -0.162 | 0.067 | 0.110 | 0.016 | 0.202 | 0.073 |
| ESM04 | -0.465 | 0.026 | 0.505 | 0.051 | 0.259 | 0.232 | 0.217 | 0.010 | 0.187 |
| FI01 | -0.035 | -0.051 | -0.061 | 0.384 | -0.040 | -0.067 | -0.024 | 0.215 | 0.071 |
| FI02 | 0.103 | -0.050 | 0.035 | 0.879 | -0.150 | -0.042 | -0.126 | 0.004 | -0.086 |
| MH01 | -0.211 | 0.029 | 0.190 | -0.142 | 0.649 | 0.317 | 0.234 | 0.187 | 0.185 |
| MH03 | -0.222 | -0.032 | 0.220 | 0.027 | 0.666 | 0.375 | 0.266 | 0.271 | 0.237 |
| MH04 | -0.110 | 0.090 | 0.167 | -0.166 | 0.626 | 0.528 | 0.274 | 0.080 | 0.195 |
| MH05 | -0.063 | -0.057 | 0.143 | -0.063 | 0.722 | 0.447 | 0.291 | 0.112 | 0.207 |
| MH06 | -0.100 | 0.016 | 0.250 | -0.141 | 0.639 | 0.440 | 0.317 | 0.206 | 0.200 |
| MH07 | -0.213 | 0.191 | 0.267 | -0.137 | 0.629 | 0.460 | 0.244 | 0.064 | 0.230 |
| MH08 | -0.140 | -0.058 | 0.197 | 0.014 | 0.640 | 0.406 | 0.304 | 0.132 | 0.200 |
| MH09 | -0.156 | -0.072 | 0.198 | -0.081 | 0.644 | 0.348 | 0.324 | 0.082 | 0.093 |
| MH10 | -0.111 | 0.048 | -0.034 | -0.127 | 0.580 | 0.307 | 0.215 | -0.053 | 0.139 |
| MH11 | -0.218 | 0.191 | 0.291 | -0.127 | 0.699 | 0.514 | 0.291 | 0.105 | 0.286 |
| MH12 | -0.136 | -0.029 | 0.176 | -0.167 | 0.661 | 0.338 | 0.210 | 0.060 | 0.144 |
| MHW01 | -0.049 | 0.132 | 0.114 | -0.052 | 0.476 | 0.657 | 0.153 | 0.137 | 0.197 |
| MHW02 | -0.151 | 0.080 | 0.261 | -0.087 | 0.535 | 0.812 | 0.374 | -0.013 | 0.185 |
| MHW04 | -0.097 | 0.244 | 0.362 | -0.007 | 0.355 | 0.653 | 0.155 | 0.192 | 0.181 |
| PB02 | -0.195 | -0.015 | 0.011 | -0.169 | 0.272 | 0.125 | 0.504 | -0.049 | 0.111 |
| PB03 | -0.180 | -0.113 | 0.119 | 0.004 | 0.317 | 0.201 | 0.655 | 0.007 | 0.024 |
| PB04 | -0.182 | 0.011 | 0.031 | -0.044 | 0.251 | 0.321 | 0.816 | -0.016 | 0.170 |
| PE01 | -0.032 | -0.026 | 0.133 | 0.129 | 0.130 | 0.065 | -0.075 | 0.788 | 0.146 |
| PE02 | -0.068 | 0.048 | 0.206 | 0.084 | 0.174 | 0.149 | -0.032 | 0.963 | 0.145 |
| PH01 | 0.209 | 0.142 | -0.149 | 0.259 | -0.318 | -0.129 | -0.546 | 0.127 | 0.015 |
| SM01 | 0.095 | -0.041 | -0.001 | -0.138 | 0.155 | 0.114 | 0.166 | -0.065 | 0.573 |
| SM03 | -0.049 | 0.135 | 0.187 | -0.019 | 0.213 | 0.298 | 0.137 | -0.030 | 0.697 |
| SM04 | -0.107 | 0.045 | 0.153 | 0.010 | 0.251 | 0.129 | 0.027 | 0.320 | 0.787 |

Source: Survey

**Supplementary Material, Table S4:** Variance Inflation Test to Check the Multicollinearity

| Observed Variables | VIF |
| --- | --- |
| EDM00 | 1.249 |
| EDM01 | 1.431 |
| EDM02 | 1.192 |
| ESE01 | 1.335 |
| ESE02 | 1.335 |
| ESM02 | 1.179 |
| ESM03 | 1.177 |
| ESM04 | 1.012 |
| FI01 | 1.011 |
| FI02 | 1.011 |
| MH01 | 1.530 |
| MH03 | 1.651 |
| MH04 | 1.433 |
| MH05 | 1.836 |
| MH06 | 1.552 |
| MH07 | 1.441 |
| MH08 | 1.514 |
| MH09 | 1.873 |
| MH10 | 1.608 |
| MH11 | 1.604 |
| MH12 | 2.073 |
| MHW01 | 1.147 |
| MHW02 | 1.230 |
| MHW04 | 1.089 |
| PB02 | 1.177 |
| PB03 | 1.189 |
| PB04 | 1.147 |
| PE01 | 1.541 |
| PE02 | 1.541 |
| PH01 | 1.239 |
| SM01 | 1.107 |
| SM03 | 1.087 |
| SM04 | 1.093 |

Source: Survey

**Supplementary Material, Table S5:** Multicollinearity Test for Latent Variables

| Latent Variables | Empowerment Decision Making | Empowerment Self-Esteem | Empowerment Self =Management | Financial Issues | Mental Health | Mental Health Awareness | Parents Behavior | Parents Education | Social media |
| --- | --- | --- | --- | --- | --- | --- | --- | --- | --- |
| Empowerment Decision Making |  |  |  |  | 1.027 |  |  |  |  |
| Empowerment Self-Esteem |  |  |  |  | 1.050 | 1.010 |  |  |  |
| Empowerment Self-Management |  |  |  |  |  | 1.061 |  |  |  |
| Financial Issues |  |  |  |  | 1.014 |  |  |  |  |
| Mental Health |  |  |  |  |  |  |  |  |  |
| Mental Health Awareness |  |  |  |  | 1.135 |  |  |  |  |
| Parents Behavior |  |  |  |  |  | 1.023 |  |  |  |
| Parents Education |  |  |  |  |  | 1.048 |  |  |  |
| Social media | 1.000 |  |  |  | 1.076 |  |  |  |  |

Source: Survey
